# Synthesis and *In Vitro* Evaluation of a Set of 6-Deoxy-6-thio-carboranyl d-Glucoconjugates Shed Light on the Substrate Specificity of the GLUT1 Transporter

**DOI:** 10.1021/acsomega.2c03646

**Published:** 2022-08-17

**Authors:** Jelena Matović, Juulia Järvinen, Iris K. Sokka, Philipp Stockmann, Martin Kellert, Surachet Imlimthan, Mirkka Sarparanta, Mikael P. Johansson, Evamarie Hey-Hawkins, Jarkko Rautio, Filip S. Ekholm

**Affiliations:** † Department of Chemistry, University of Helsinki, P.O. Box 55, FI-00014 Helsinki, Finland; ‡ School of Pharmacy, University of Eastern Finland, P.O. Box 1627, FI-70211 Kuopio, Finland; § Institute of Inorganic Chemistry, Leipzig University, D-04103 Leipzig, Germany; ∥ Helsinki Institute of Sustainability Science, HELSUS, FI-00014 Helsinki, Finland; ⊥ CSC − IT Center for Science Ltd., P.O. Box 405, FI-02101 Espoo, Finland

## Abstract

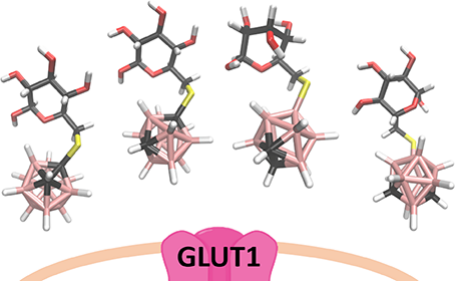

Glucose- and sodium-dependent glucose transporters (GLUTs and SGLTs) play vital roles in human biology. Of the 14 GLUTs and 12 SGLTs, the GLUT1 transporter has gained the most widespread recognition because GLUT1 is overexpressed in several cancers and is a clinically valid therapeutic target. We have been pursuing a GLUT1-targeting approach in boron neutron capture therapy (BNCT). Here, we report on surprising findings encountered with a set of 6-deoxy-6-thio-carboranyl d-glucoconjugates. In more detail, we show that even subtle structural changes in the carborane cluster, and the linker, may significantly reduce the delivery capacity of GLUT1-based boron carriers. In addition to providing new insights on the substrate specificity of this important transporter, we reach a fresh perspective on the boundaries within which a GLUT1-targeting approach in BNCT can be further refined.

## 1 Introduction

Glucose- and sodium-dependent glucose transporters (GLUTs and SGLTs) play a central role in human biology.^1,2^ They are responsible for transporting the vital energy source d-glucose across the plasma membrane, which is essential for the sustenance of living cells. Therefore, glucose transporters are expressed on all cells. This makes them appealing targets from a molecular biology perspective. These transporters can be harnessed for multiple purposes and have been considered as promising therapeutic targets for prodrug strategies for a considerable timespan.^3−5^ While this is true for most transporters belonging to these families, the GLUT1 transporter has become the central target in the cancer research field, as it is overexpressed on a variety of cancers.^6^ In addition, the impaired d-glucose metabolism and increased d-glucose demand observed in cancer cells further increase the appeal of this approach.^7,8^ Indeed, the potential embedded in a GLUT1-targeting approach within the cancer research field is already clear as this targeting strategy is in widespread clinical use as a tumor imaging technique (2-deoxy-2-fluoro-d-glucose (FDG) in combination with positron emission tomography imaging).^9^ Due to the existing sound foundations, pursuing a GLUT1-targeting strategy within a boron neutron capture therapy (BNCT) frame represents an interesting possibility.

**Figure 1:**
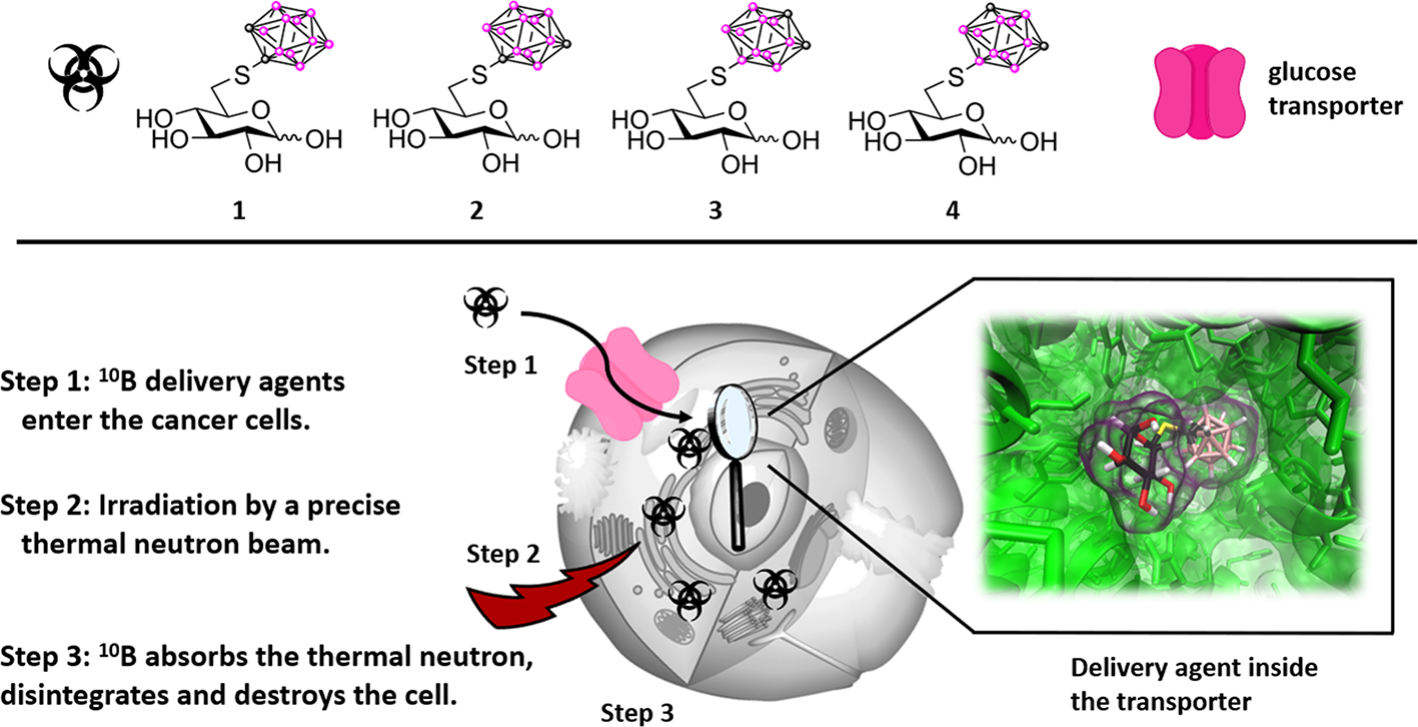
Top: Chemical structures of the four 6-deoxy-6-thio-carboranyl d-glucoconjugates synthesized and assessed and indication of symbols used in the figure below (boron atoms in pink and carbon atoms in gray in the boron cluster). Bottom: Principles of BNCT. The ^10^B delivery agents enter a cancer cell through GLUT1 (Step 1), and the cell is then irradiated with a precise thermal neutron beam (Step 2). ^10^B captures the thermal neutrons briefly forming ^11^B*. Excited ^11^B* quickly undergoes a fission reaction producing ^4^He and ^7^Li. ^4^He nuclei have a destructive effect on the cell (Step 3).

In a BNCT context, the transportation speed and capabilities of GLUT1 can be harnessed to deliver boron-10 atoms across the plasma membrane into the cancer cells. Once the intracellular boron concentration reaches 20–35 μg/g of tumor and a high tumor-to-blood/tumor-to-normal tissue ratio is observed (above 3:1 but ideally more than 10:1), the cancer cells can in theory be selectively eradicated in a separate step by irradiation with an external thermal neutron beam at the tumor site (see Figure 1).^10−12^ Recently, we have been exploring the premises of a GLUT1-targeting strategy for BNCT in detail, thereby complementing the earlier work conducted in the field.^13,14^ Through our medicinal chemistry approach, we have been able to identify the most promising boron cluster attachment site in d-glucose and our comprehensive *in vitro* assessment proved that on the molecular biology level, the GLUT1-targeting strategy is able to outperform the LAT1-targeting and passive transport strategies in current clinical use (boronophenylalanine (BPA)^15^ and sodium borocaptate (BSH)^16^). Therefore, the continued refinement of the GLUT1-targeting strategy is warranted. Herein, we set out to shorten the synthetic routes to these types of boron delivery agents by switching the boron cluster conjugation strategy and attachment points while continuing to build a deeper overall understanding of the biochemical foundations of this approach. A representative library of 6-deoxy-6-thio-carboranyl d-gluconjugates were synthesized and characterized in detail by a wide palette of NMR spectroscopic techniques and HRMS before being subjected to a preliminary *in vitro* assessment featuring cytotoxicity, experimental and computational GLUT1 affinity, and cellular uptake studies. Notable differences to our earlier work were observed.^13,14^ In more detail, subtle structural modifications in the carborane and linker structures were found to significantly diminish the delivery capacity of these boron carriers. These results help identify the boundaries within which the GLUT1-targeting approach to BNCT can be further refined and aid in the interpretation of earlier observations noted in the field.^17−20^

## 2 Results and Discussion

### 2.1 Synthesis and Characterization

**Scheme 1:**
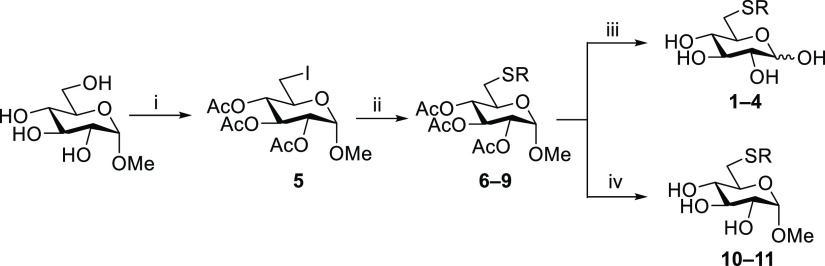
Synthetic Routes to **1**–**4**, **10**, and **11** Reaction conditions: (i) (1) PPh_3_, imidazole, I_2_, toluene, 80 °C, 1 h; (2) Ac_2_O:pyridine 1:1, rt, 17 h, 63%; (ii) corresponding mercapto-carborane, *N*,*N*-diisopropylethylamine (DIPEA) or K_2_CO_3_, acetone, 60 °C, 16–24 h, 55% (**6**, R = 1-*o*Cb), 98% (**7**, R = 1-*m*Cb), 80% (**8**, R = 9-*o*Cb), 90% (**9**, R = 9-*m*Cb); (iii) 1–4 N HCl, 110–120 °C, 4–7 h, 35% (**1**, R = 1-*o*Cb), 43% (**2**, R = 1-*m*Cb), 75% (**3**, R = 9-*o*Cb), 74% (**4**, R = 9-*m*Cb); (iv) 1 N HCl, 85–105 °C, 2–4 h, 84% (**10**, R = 1-*m*Cb), 74% (**11**, R = 9-*m*Cb).

The first report on the synthesis of carbohydrate-based delivery agents for BNCT was reported by Hawthorne *et al.* in 1988.^21^ Since then, a vast number of glycoconjugates has been synthesized for this purpose.^19^ Not only have different targeting strategies been pursued,^14,22,23^ the carbohydrates have at times likewise been employed in order to modify the physicochemical properties of other delivery agents.^23,24^ One of the pursued targeting strategies, which holds tremendous potential, is the GLUT1-targeting approach. Unfortunately, insights on the biochemical foundations and boundaries of this approach are limited, as detailed studies focusing on these central aspects have only recently begun to emerge.^13,14,25^ In our recent work, we synthesized and studied the toxicity, GLUT1-affinity, and cellular uptake profiles of the complete positional isomer library of *ortho*-carboranylmethyl-bearing d-glucoconjugates.^13,14^ We revealed that the optimal attachment site for a boron cluster in the carbohydrate core is position 6. In the current study, we set out to shorten the synthetic routes to GLUT1-based boron carriers by targeting d-glucoconjugates bearing an *S*-linked carboranyl substituent at position 6. We envisioned that the nonionic nature of the boron cluster selected, in combination with the elongated bond lengths to sulfur, would allow us to preserve the functional basis of these delivery agents. In addition, we decided to map the effects of the attachment site and configuration of the boron cluster by preparing a representative set containing both *ortho*- and *meta*-carboranes (1,2-dicarba-*closo*-dodecaborane (-*o*Cb) and 1,7-dicarba-*closo*-dodecaborane (-*m*Cb), respectively), connected to sulfur through either a carbon or a boron atom (1-Cb and 9-Cb, respectively). From a synthesis perspective, the *S*-linked carboranyl cluster is commonly installed through an S_N_2-displacement reaction regardless of the attachment site or structure of the carborane.^26−28^ Therefore, we envisioned that a protected 6-deoxy-6-iodo glucopyranoside building block could serve as a suitable carbohydrate-based electrophile. The synthetic routes to the six end products **1**–**4**, **10**, and **11** are summarized in Scheme 1 and will be discussed in more detail next.

While the carbohydrate starting material **5** is commercially available, it can likewise be prepared from methyl α-d-glucopyranoside in two synthetic steps.^29,30^ We opted to utilize this methyl glucoside as a starting material since this would allow both the synthesis of a pure anomer as well as the more important hemiacetal through the same synthetic sequence. The S_N_2-displacement reaction was performed according to literature protocols with four different carboranyl thiols (1-*o*Cb, 1-*m*Cb, 9-*o*Cb, and 9-*m*Cb) under slightly basic conditions in acetone.^26,27^ The isolated yields of **1**–**4** were in the 55–98% range. While the reaction conditions were not optimized, the yields are nevertheless competitive as, e.g., installing a carboranyl species through a coupling reaction between decaborane and an alkyne usually results in maximum yields of 65%.^31^

With the protected glucoconjugates at hand, we decided to evaluate whether the deprotection conditions could be tailored to access either the fully deprotected hemiacetal or its methyl glucoside. The most commonly employed deacetylation conditions, i.e., Zemplén conditions,^32^ would not be suitable for this task as certain carboranyl species are labile to basic conditions.^33^ Therefore, we decided to explore the possibility of using acidic conditions instead. Aqueous hydrochloric acid solutions at elevated temperatures have been reported to work well for this purpose.^34^ In our early trials, the solubility of the protected glucoconjugates was low under the employed conditions and therefore elevated temperatures were a necessity.

Simultaneously, it should be mentioned that we have observed a difference in the aqueous solubility between the carbon- (**1**–**2**) and boron-linked carboranes (**3**–**4**), with the boron-linked species displaying an enhanced solubility compared to their carbon-linked counterparts. Regardless of these factors, we found that the deprotection outcome could be successfully altered by employing a 1–4 M solution of HCl while varying the reaction temperature and time. In more detail, employing temperatures in the 85–105 °C range and reaction times up to 4 h resulted in the formation of the deacetylated methyl glucopyranosides. However, increasing the temperature to the 115–120 °C range and extending the reaction time by a few hours resulted in the hydrolysis of the methyl glycoside. While all four fully deprotected hemiacetals could be obtained through this protocol, notable differences in the yields were observed. Substantially higher yields were obtained with clusters linked through a boron atom (73–75%) than for their carbon-linked counterparts (35–43%). We evaluated a number of different acids and conditions (trifluoroacetic acid (TFA), HCl, different co-solvents, etc.); however, only minor differences were noted in the isolated yields. Thus, no further emphasis was put on exploring the underlying reasons for these observations as short and accessible synthetic routes to the new types of GLUT1-based delivery agents had been developed.

**Figure 2:**
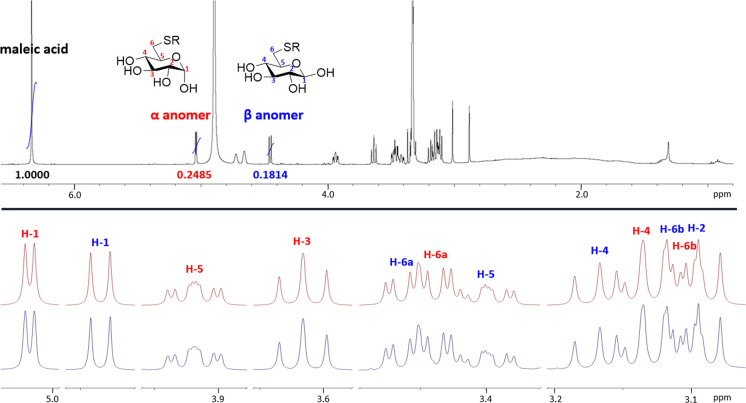
Highlights from the NMR spectroscopic characterization of compound **1**. Top: qNMR spectrum of **1** (R = 1-*o*Cb) using maleic acid as the internal standard. Bottom: 5.0–3.0 ppm region of the ^1^H NMR spectrum of compound **1** showcasing the accuracy of the spectral simulation (simulated spectrum at the top, measured spectrum at the bottom).

During the synthetic work, NMR spectroscopic (nuclear magnetic resonance, variety of 1D/2D-techniques) and mass spectrometric techniques (high-resolution mass spectrometry, HRMS) were utilized to ascertain the molecular structures of the compounds and quantitative NMR (qNMR) was employed in determining the purity of the final products **1**–**4** prior to their *in vitro* assessment. The NMR spectra were fully assigned using 1D-TOCSY (total correlation spectroscopy), ^1^H, ^13^C{^1^H}, ^11^B{^1^H}, COSY (correlation spectroscopy), ed-HSQC (edited heternonuclear single quantum coherence), and HMBC (heteronuclear multiple bond correlation). The ^1^H NMR spectra were further simulated with the ChemAdder software.^35,36^ We use **1** as an example to shortly convey the process used. Highlights from the NMR spectroscopic characterization are provided in Figure 2. Due to mutarotation, **1** exists as a mixture of anomers. Using ed-HSQC, the C-1 atoms, at 98.3 and 94.0 ppm, and the corresponding H-1 atoms, at 5.04 and 4.45 ppm, could easily be identified. The β anomer, at 4.45 ppm, has a distinctly higher coupling constant (*J*_1,2_ = 7.8 Hz) due to the larger dihedral angle between the axial H-1 and H-2 protons. The α anomer, at 5.04 ppm, has a smaller coupling constant of (*J*_1,2_ = 3.7 Hz) corresponding to an axial-equatorial relationship between H-1 and H-2. Through the conventional use of COSY, ed-HSQC, and HMBC, most of the signals can be identified; however, in severely overlapping regions of the ^1^H NMR spectrum, this becomes increasingly challenging. To overcome these issues, we did spectral simulation work with the ChemAdder software and were able to obtain accurate coupling constants and chemical shifts for all signals, including those in crowded areas of the spectrum (see Figure 2). In addition, we used the ChemAdder software to determine the anomeric ratios, which were found to be similar for compounds **1**–**4** (α:β 56:44 for **1**, 49:51 for **2**, 48:52 for **3**, and 55:45 for **4**). Finally, qNMR was utilized to ascertain that the purity of the compounds (**1**–**4**) submitted for the *in vitro* assessment studies exceeded 95%. The protocol described by Pauli *et al.*(37) was employed, and maleic acid was used as an internal standard.

### 2.2 Molecular Recognition Studies

**Figure 3:**
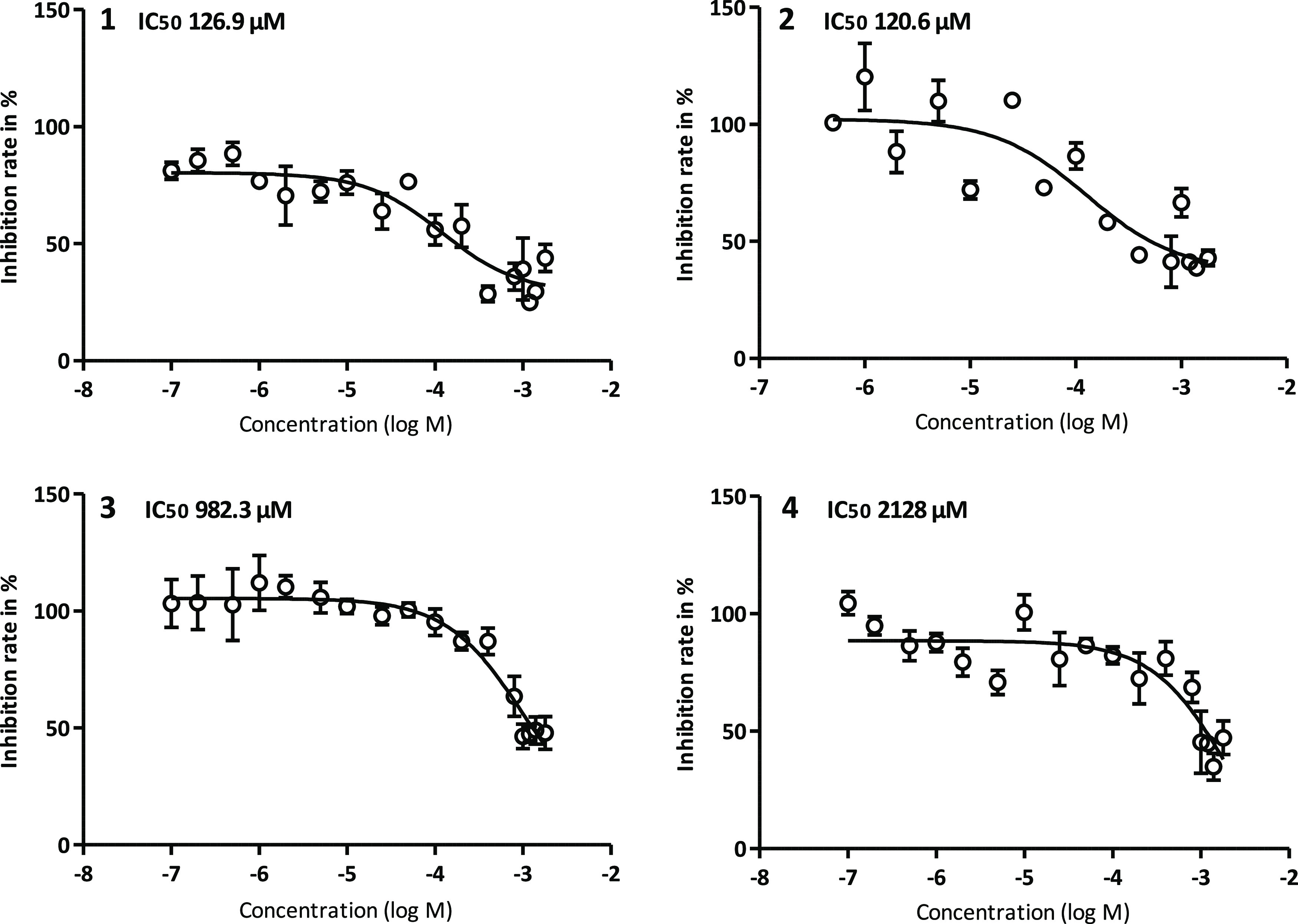
The affinity curves and the calculated IC_50_ values obtained through the *cis*-inhibition assays are displayed for each of the four glucoconjugates **1**, **2**, **3**, and **4**.

From the drug development perspective, it is central to understand the molecular interactions between the ligand and the transporter. For example, in a BNCT context, the selectivity and success of the treatments are directly related to the preferential uptake of boron-10 enriched delivery agents by cancer cells. In our case, the GLUT1 transporter, which is overexpressed on various cancers, is responsible for the uptake process. The clinical validity and therapeutic utility of the approach stem from the “Warburg effect”.^7,8^ Cancer cells have an impaired d-glucose metabolism while simultaneously displaying a high proliferation rate and energy demand. Together, these factors contribute to an increased d-glucose uptake in cancer cells compared to healthy cells and offer a basis for pursuing a GLUT1-targeting strategy in BNCT. Based on the available tumor imaging data obtained through the use of FDG,^38^ head and neck cancers represent a promising target for our approach. Therefore, we employed the human squamous carcinoma cell line CAL 27 as a suitable model for the *in vitro* assessment of GLUT1-targeting delivery agents. In addition to being a relevant head and neck cancer cell line of human origin, the GLUT1-transporter has been indicated to play a key role in the aberrant growth of CAL 27 cells.^39,40^ In our previous work, we validated the GLUT1-function, quantified the GLUT1-expression, and developed a *cis*-inhibition assay for determining the relative affinities of GLUT1-targeting delivery agents to GLUT1 in the CAL 27 cell line.^13,14^ To allow comparison of the results of the present study to our earlier ones, we used the same protocols herein. The fully deprotected glucoconjugates **1**–**4** were subjected to the *cis*-inhibition assay, i.e., they were forced to compete for the transporter against radiolabeled [^14^C]-d-glucose in order to assess their targeting capabilities from a functional point of view. A high GLUT1 affinity, i.e., a low IC_50_ value, is characteristic of delivery agents that are able to target the transporter in the presence of the natural substrate d-glucose in a biological milieu. The experimentally determined IC_50_ values were 126.9 μM for **1**; 120.6 μM for **2**; 982.3 μM for **3**; 2128 μM for **4**; and >1000 μM for the control d-glucose (Figure 3). Large differences in the IC_50_ values were thus noted. While the evaluated library is limited, a clear trend pointing toward the importance of the cluster conjugation site was revealed. The carbon-linked species **1**–**2** displayed GLUT1 affinities that were only slightly lower than those previously reported for the *O*-carboranylmethyl-bearing positional isomer library.^13,14^ These two delivery agents display acceptable GLUT1-targeting capabilities. The boron-linked species **3**–**4** on the other hand displayed significantly lower affinities to the GLUT1-transporter, indicating that the targeting capabilities are not sufficient for use as boron delivery agents in BNCT. While the trend is clear, the results are surprising. The question that arises is as follows: which underlying factors contribute to these notable deviations uncovered through the experimental *cis*-inhibition assay?

In an attempt to provide an answer to this intriguing question, we set out to inspect the molecular interactions between GLUT1 and the delivery agents through a docking study. We used our previously benchmarked computational model.^13,14^ Our model is derived from XylE, a d-xylose-proton symporter, for which both the inside open (PDB ID 4QIQ)^41^ and outside open (PDB ID 6N3I)^42^ crystal structures are available. This transporter shares a structural similarity with the GLUT1–4 proteins (29% sequence identity and 49% similarity),^43^ the carbohydrate-binding domain is well preserved, and by virtual mutation of a single amino acid residue, Gln-415 to Asn-415, a structurally similar binding pocket to that in GLUT1 can be constructed.^44,45^ The carbohydrate delivery agents exist as a mixture of anomers; however, the individual anomers were initially modeled separately in the docking assay and then the overall mean binding energies were calculated to fit with the experimentally determined anomeric ratios. While the mean binding energies were calculated for both the outside and inside open conformations, the outside open conformation is more important when forming a tie to the experimental affinity data. Based on the docking study, the species containing carbon-linked clusters interact more strongly with GLUT1 than the species that contain boron-linked clusters. The computational results support the experimental ones, although the differences in mean binding energies are not as striking as the experimentally determined values would suggest. They do fall within the experimental margin of error, i.e., 1 kcal/mol. In our earlier work,^13,14^ the computational model has been found to be a reliable indicator of observed GLUT1 affinity and thus great care needs to be practiced when interpreting the overall results obtained. Two possibilities for the slight discrepancy come to mind: (1) the limitations of the current docking model, which does not take molecular dynamic factors into account on a sufficient level, and/or, (2) the boron-linked clusters display deviating overall properties in a biological milieu compared to the carbon-linked cluster and these hinder/limit their ability to compete for the transporter. Based on the results generated, it is too early to draw definite conclusions and more work with a larger substrate library will be required to map the molecular level basis of the observations noted. In order to gather more pieces of the puzzle, we continued our *in vitro* assessment by evaluating the cytotoxicity and uptake profiles of compounds **1**–**4**.

### 2.3 Cytotoxicity and Boron Delivery Capacity

With the observations noted in our molecular recognition studies, we proceeded with the *in vitro* assessment. We chose to focus on two topics that are of interest from a potential translational medicine perspective: cytotoxicity and boron delivery capacity. These studies were performed in the CAL 27 cell line in order for the results to be comparable to our earlier work.^13,14^ The importance of being able to compare results across series of molecules cannot be sufficiently stressed when aiming to understand the biochemical premises of the GLUT1-approach. On a general level, the delivery agents need to display low cytotoxicity and a high boron delivery capacity; otherwise, they will not be suitable for clinical use.

**Figure 4:**
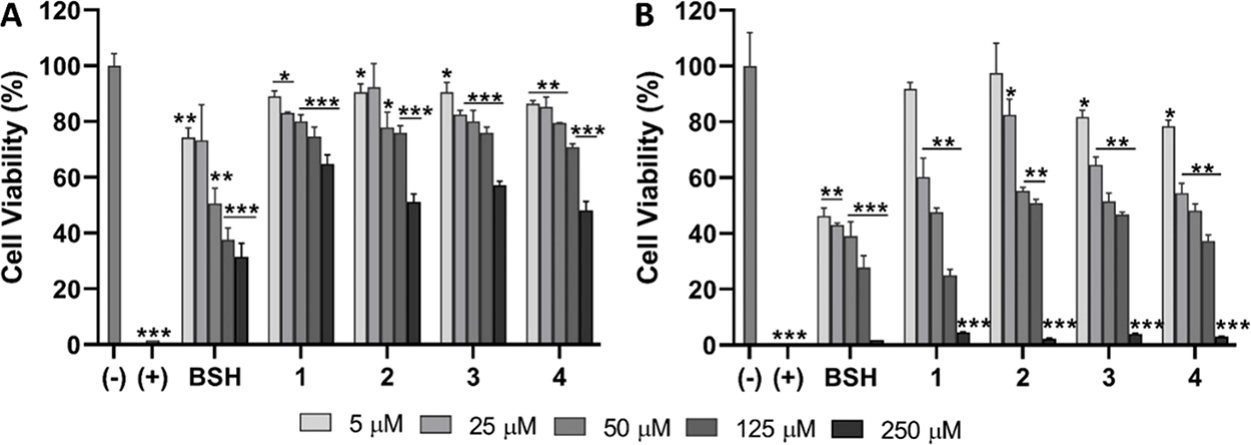
Results from the cytotoxicity studies of **1**–**4** and BSH across the 5–250 μM concentration range in the CAL 27 cell line. The incubation times were 6 h (A) and 24 h (B). The cell culture medium was used as the negative control and 1% Triton X-100 as the positive control. The statistical significance was determined through an unpaired Student’s *t*-test where the significance was set at *p < 0.05, **p < 0.01, and ***p < 0.001.

In the cytotoxicity assays, a series containing the glucoconjugates **1**–**4**, BSH (as a representative of a clinically approved boron delivery agent), non-ionic surfactant triton X (as a positive control for cytotoxicity), and the cell culture media (as a negative control) were screened. The concentration range (5–250 μM) and time points (6 and 24 h) were selected based on our previous experiences from working with the GLUT1-targeting approach.^13,14^ A commercial CellTiter-Glo assay was used to quantify cell viability based on the ATP production of the live cells after treatments. The results are summarized in Figure 4. It is worth mentioning that the substantial differences in the experimental GLUT1 affinities noted do not influence the cytotoxicity profiles of these delivery agents. Regardless of time points and concentrations employed, the investigated glucoconjugates appear to be less toxic than the clinically employed boron carrier BSH. This entails that the toxicity of these agents would not hamper their use in BNCT, although it should be mentioned that a complete view of the toxicity profiles is not possible to obtain through *in vitro* studies alone. A notable difference in the cytotoxicity between the 6 h and 24 h time points was observed. At the 24 h time point, the glucoconjugates **1**–**4** were considerably more toxic than the corresponding glucoconjugates bearing an *O*-linked *ortho*-carboranylmethyl substituent at position 6.^14^ Therefore, the *O*-linked *ortho*-carboranylmethyl substituent seems to be better tolerated than the *S*-linked carboranyl substituent in the GLUT1-targeting delivery agents. On a general level, the toxicity profiles are likely to be connected to a certain extent to the uptake profiles. Therefore, as a last measure, we sought to assess the boron delivery capacity of the synthesized glucoconjugates **1**–**4**. In these studies, the GLUT1-targeting strategy was further compared to the clinically employed delivery agents BPA and BSH, which enter cancer cells either through a LAT1-mediated uptake (BPA) or a passive diffusion through the cell membrane (BSH).

**Figure 5:**
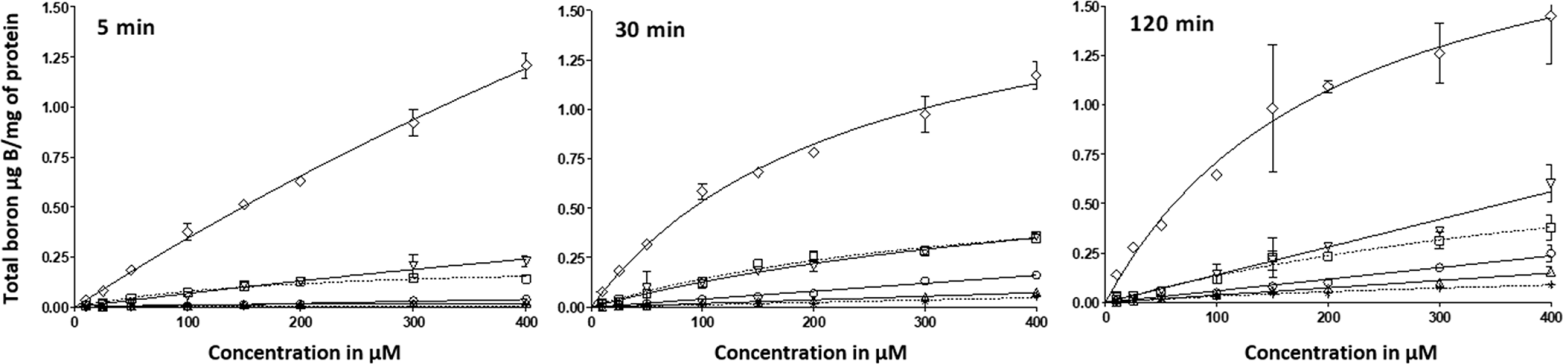
Results from cellular uptake studies performed in the CAL 27 cell line. The following substrates were included: **1** (circle), **2** (diamond), **3** (triangle), **4** (inverted triangle), BPA (square), and BSH (asterisk) across the 10–400 μM concentration range. The incubation times were 5, 30, and 120 min. (*n* = 3 at all three time points). The following Michaelis–Menten kinetic parameters were obtained (*V*_max_ is given as μg B/mg protein; *K*_m_ is given as μM) at 5 min incubation time, **1**: *V*_max_ = 1.34; *K*_m_ = 1827.8, **2**: *V*_max_ = 6.60; *K*_m_ = 1813, and **3**: *V*_max_ = 0.03; *K*_m_ = 109.7. At 30 min incubation time, **1**: *V*_max_ = 0.83; *K*_m_ = 555.6, **2**: *V*_max_ = 1.80; *K*_m_ = 236.9. At 120 min incubation time, **2**: *V*_max_ = 2.19; *K*_m_ = 207.6.

The uptake studies were performed according to our recently reported protocol.^13,14^ It should be noted that the protocol employed does not differentiate the intracellular boron concentration from that arising from delivery agents trapped on the cell membrane. This, however, is not a concern, as the cell-killing effect caused by the thermal neutron beam in actual BNCT treatments has a destructive radius reminiscent to the diameter of a single cell (5–9 μm in biological tissue).^46^ In the uptake studies, the concentration range 10–400 μM was screened. The incubation times 5, 30, and 120 min were chosen due the optimal performance of [^14^C]-d-glucose under these conditions. Since inductively-coupled plasma mass spectrometry (ICP-MS) is somewhat insensitive when it comes to quantification of the boron content, thoroughly washed cells from four wells were combined and digested before being subjected to the analysis. The boron quantification results are presented in Figure 5. The new set of glucoconjugates was found to display comparable boron delivery capacity to the delivery agents in current clinical use (BPA and BSH) with the exception of glucoconjugate **2**, which has a fivefold higher delivery capacity compared to the rest of the delivery agents screened. However, when these results are compared to the ones obtained with our previously reported hit molecule 6-*O*-carboranylmethyl d-glucose,^14^ the *S*-linked carboranyl species seem to be at a significant disadvantage as their boron delivery capacity is roughly 5–100 times lower. However, we noticed that the *K*_m_ values were consistently higher with the *S*-linked carboranyl species compared to the *O*-linked carboranylmethyl species.^14^ The higher *K*_m_ value may imply that there are additional transport mechanisms involved in the uptake of the *S*-series delivery agents. More work is needed to ascertain these factors. Regardless, the different *K*_m_ values may likewise indicate that a greater concentration, which may no longer be clinically relevant, would be required for the *S*-series species to reach *V*_max_.

When trying to form a connection between the GLUT1-affinity and the cellular uptake profiles, it is important to note that the affinity results convey the competitive edge of the glucoconjugates over the natural substrate in terms of targeting GLUT1, whereas the uptake profiles showcase the ability of the glucoconjugates to remain attached to or within the cells. Therefore, we did not expect the affinity results to correlate directly with the uptake profiles. The large deviations in the uptake profiles, especially in light of our previous results, are nevertheless very interesting. Based on the results, glucoconjugate **1** seems to act as a GLUT1-antagonist while glucoconjugate **2** still displays acceptable properties from the boron delivery perspective. In more detail, both agents display high binding affinity to GLUT1; however, only glucoconjugate **2** enters the cells through the transporter at an acceptable rate. These findings highlight that great diligence should be practiced when switching atoms in the carbohydrate core and may explain, for example, why the previously prepared BSH-glucose conjugates have been discarded at an early development stage.^20^ Altogether, our findings provide new insights on the substrate specificity of the important GLUT1-transporter and will aid in the rational design of therapeutic approaches focusing on this target.

## 3 Conclusions

We are currently developing a GLUT1-targeting strategy for BNCT of head and neck cancers. The first phase of our medicinal chemistry approach has been devoted to exploring the biochemical foundations in detail. Recently, we reported on new types of GLUT1-based delivery agents that were able to outshine the clinically employed boron carriers in a detailed *in vitro* assessment.^13,14^ Herein, we attempted to shorten the synthetic routes to such delivery agents while simultaneously preserving their functional basis. We developed short and accessible synthetic methods for the construction of a set of 6-deoxy-6-thio-carboranyl d-glucoconjugates and characterized the products and all intermediates by a wide palette of NMR spectroscopic techniques and mass spectrometry. Four glucoconjugates were subjected to a preliminary *in vitro* assessment including molecular recognition, cytotoxicity, and cellular uptake studies. The molecular recognition studies revealed that the atom involved in cluster conjugation has a marked effect on the GLUT1 affinity; species connected to a carbon atom have a significantly higher affinity than species connected to a boron atom. While qualitatively agreeing with the experimental observations, the molecular basis of these observations could not be completely uncovered with our current computational model. More work in this area including considerations on dynamics and more accurate descriptions of intermolecular interactions is needed to fully understand the underlying factors at play.

All four glucoconjugates displayed acceptable cytotoxicity profiles and their boron delivery capacity was found to be in the same range as those of the clinically employed agents BPA and BSH, apart from glucoconjugate **2**, which was found to be roughly five times more efficient. However, the boron delivery capacity was found to be significantly lower compared to our previous molecular libraries,^13,14^ and especially, glucoconjugate **1** seemed to display a GLUT1 antagonist behavior rather than act as a promising delivery agent for BNCT. In addition to providing knowledge that may help explain earlier observations noted in the field,^17−20^ our results provide understanding on the boundaries within which this approach to BNCT can be further developed. The new insights on the substrate specificity of the GLUT1 transporter are important for the development of other therapeutic approaches centered on this molecular biology target. Overall, the results point toward the dire need of continued studies on the biochemical foundations of GLUT1-targeting strategies with more diverse substrate libraries, a topic that we are currently in full pursuit of.

## 4 Experimental Section

### 4.1 Synthesis and Structural Characterization Data

All starting materials and reagents were commercially purchased and used without further purification. Solvents used in reactions were purified with the VAC vacuum solvent purification system and further dried over 4 Å molecular sieves. Reactions were carried out under inert conditions using either an argon or nitrogen atmosphere. For all NMR experiments, a Bruker Avance III spectrometer was used (operating at ^1^H: 500.13 MHz, ^13^C: 125.76 MHz, ^11^B: 160.46 MHz) and the probe temperature was kept at 25 °C. All intermediates as well as final products were fully characterized using 1D (^1^H, 1D-TOCSY, ^13^C{^1^H}, and ^11^B{^1^H}) and 2D (COSY, ed-HSQC, and HMBC) NMR experiments with pulse sequences provided by the instrument manufacturer. Spectral simulations were performed with the ChemAdder software in order to obtain precise chemical shifts and coupling constants. The coupling constants are reported in Hz and provided when first encountered. Coupling patterns are given as s (singlet), d (doublet), dd (doublet of a doublet), etc. Chemical shifts are expressed on the δ scale (in ppm). The following reference signals are employed: TMS (tetramethylsilane), residual chloroform, methanol, or 15% BF_3_ in CDCl_3_. HRMS were obtained with a Bruker Micro Q-TOF with electrospray ionization operated in positive mode. The purity of substrates **1**–**4** was determined to be >95% by qNMR. TLC was performed on aluminum sheets precoated with silica gel 60 F254 (Merck), and spots were uncovered by spraying with conc. H_2_SO_4_:MeOH (1:5) solution followed by heating. Compounds were purified by flash chromatography using silica gel 40 as the stationary phase.

#### 4.1.1 Substrate-Specific Analytical Data

##### 4.1.1.1 Methyl 2,3,4-Tri-*O*-acetyl-6-deoxy-6-iodo-α-d-glucopyranoside (**5**)

Synthesized over two steps, starting from methyl α-d-glucopyranoside (5.00 g, 25.8 mmol, 1.00 equiv), which was dissolved in toluene (100 mL) and heated to 80 °C. PPh_3_ (8.35 g, 31.8 mmol, 1.2 equiv) and imidazole (6.51 g, 95.6 mmol, 3.7 equiv) were added to the reaction mixture and left to stir for 10 min. I_2_ (9.96 g, 39.2 mmol, 1.5 equiv) was then added to the reaction mixture over the course of 0.5 h after which the mixture was left to reflux for 1 h. The reaction mixture was then brought to room temperature, and the product was extracted with water (3 × 100 mL). The crude product was dried, then dissolved in Ac_2_O:pyridine 1:1 (100 mL), and left to stir overnight. The solvents were then removed under reduced pressure, and the crude product was purified by column chromatography (EtOAc:Hex 1:1). Compound **5** was obtained as a white solid (6.95 g, 16.1 mmol, 63%). TLC: *R_f_*: 0.60 (EtOAc:Hex 1:1).

^1^H NMR (500.13 MHz, CDCl_3_, 25 °C): δ = 5.47 (dd, 1H, *J*_2,3_ = 10.3, *J*_3,4_ = 9.3 Hz, H-3), 4.96 (d, 1H, *J*_1,2_ = 3.7 Hz, H-1), 4.89 (dd, 1H, H-2), 4.87 (dd, 1H, *J*_4,5_ = 9.8 Hz, H-4), 3.79 (ddd, 1H, *J*_5,6a_ = 2.5, *J*_5,6b_ = 8.3 Hz, H-5), 3.48 (s, 3H, 1-OC**H**_**3**_), 3.30 (dd, 1H, *J*_6a,6b_ = −10.9 Hz, H-6a), 3.14 (dd, 1H, H-6b), 2.08 (s, 3H, 2-OCOC**H**_**3**_), 2.06 (s, 3H, 4-OCOC**H**_**3**_), and 2.01 (s, 3H, 3-OCOC**H**_**3**_) ppm.

^13^C{^1^H} NMR (125.76 MHz, CDCl_3_, 25 °C): δ = 170.2 (2-O**C**OCH_3_), 170.1 (3-O**C**OCH_3_), 169.8 (4-O**C**OCH_3_), 96.8 (C-1), 72.6 (C-4), 71.0 (C-2), 69.8 (C-3), 68.8 (C-5), 55.9 (1-O**C**H_3_), 20.8 (2-OCO**C**H_3_, 3-OCO**C**H_3_ and 4-OCO**C**H_3_), and 3.7 (C-6) ppm.

HRMS *m*/*z*: calcd for C_13_H_19_INaO_8_ [M + Na]^+^, 453.0022; found, 453.0125.

##### 4.1.1.2 Methyl 2,3,4-Tri-*O*-acetyl-6-deoxy-6-thio-(1,2-dicarba-closo-dodecaboran-1-yl)-α-d-glucopyranoside (**6**)

Compound **5** (0.66 g, 1.53 mmol, 1.00 equiv) and 1-(mercapto)-1,2-dicarba-*closo-*dodecaborane(12) (0.41 g, 2.33 mmol, 1.50 equiv) were placed in an oven-dried Schlenk flask under an inert atmosphere. The mixture was suspended in dry acetone (25 mL), and K_2_CO_3_ (0.72 g, 5.21 mmol, 3.41 equiv) was added. The reaction mixture was allowed to stir for 24 h at 60 °C. After cooling to room temperature, the pH was adjusted to 7 using 1 M HCl. Acetone was removed under reduced pressure, and the resulting aqueous solution was extracted with ethyl acetate (3 × 30 mL). The combined organic layers were washed with water and brine (30 mL) and dried over magnesium sulfate. The crude product was purified by column chromatography (EtOAc:Hex 2:3). Compound **6** was obtained as a colorless oil (0.40 g, 0.84 mmol, 55%). TLC: *R_f_*: 0.65 (EtOAc:Hex 2:3).

^1^H NMR (500.13 MHz, CDCl_3_, 25 °C) δ = 5.45 (dd, 1H, *J*_2,3_ = 10.3, *J*_3,4_ = 9.3 Hz, H-3), 4.92 (dd, 1H, *J*_4,5_ = 9.9 Hz, H-4), 4.88 (d, 1H, *J*_1,2_ = 3.6 Hz, H-1), 4.82 (dd, 1H, H-2), 3.95 (ddd, 1H, *J*_5,6a_ = 2.8, *J*_5,6b_ = 8.2 Hz, H-5), 3.78 (br s, 1H, cluster-C**H**), 3.40 (s, 3H, 1-OC**H**_**3**_), 3.13 (dd, 1H, *J*_6a,6b_ = −13.5 Hz, H-6a), 3.01 (dd, 1H, H-6b), 2.09 (s, 3H, 4-OCOC**H**_**3**_), 2.06 (s, 3H, 2-OCOC**H**_**3**_), 2.00 (s, 3H, 3-OCOC**H**_**3**_), and 3.15–1.54 (br m, 10H, cluster-B**H**) ppm.

^13^C{^1^H} NMR (125.76 MHz, CDCl_3_, 25 °C) δ = 170.3, 170.1, 170.0 (2-O**C**OCH_3_, 3-O**C**OCH_3_ and 4-O**C**OCH_3_), 96.8 (C-1), 74.5 (cluster-**C**), 71.5 (C-4), 70.8 (C-2), 69.8 (C-3), 68.8 (cluster-**C**H), 68.2 (C-5), 55.8 (1-O**C**H_3_), 38.5 (C-6), and 20.8 and 20.7 (2-OCO**C**H_3_, 3-OCO**C**H_3_ and 4-OCO**C**H_3_) ppm.

^11^B{^1^H} NMR (160.46 MHz, CDCl_3_, 25 °C) δ = −1.6, −4.9, −8.7, −10.5, −12.6 ppm.

HRMS: *m*/*z* calcd. for B_10_C_15_H_30_NaO_8_S [M + Na]^+^: 503.2490, found: 503.2494.

##### 4.1.1.3 Methyl 2,3,4-Tri-*O*-acetyl-6-deoxy-6-thio-(1,7-dicarba-closo-dodecaboran-1-yl)-α-d-glucopyranoside (**7**)

Compound **5** (0.65 g, 1.51 mmol, 1.00 equiv) and 1-(mercapto)-1,7-dicarba-*closo-*dodecaborane(12) (0.40 g, 2.27 mmol, 1.50 equiv) were placed in an oven-dried Schlenk flask under an inert atmosphere. The mixture was suspended in dry acetone (25 mL), and K_2_CO_3_ (0.63 g, 4.54 mmol, 3.00 equiv) was added. The reaction mixture was allowed to stir for 24 h at 60 °C. After cooling to room temperature, the pH was adjusted to 7 using 1 M HCl. Acetone was removed under reduced pressure, and the resulting aqueous solution was extracted with ethyl acetate (3 × 30 mL). The combined organic layers were washed with water and brine (30 mL) and dried over magnesium sulfate. The crude product was purified by column chromatography (EtOAc:Hex 1:2). Compound **7** was obtained as a colorless foam (0.72 g, 1.48 mmol, 98%). TLC: *R_f_*: 0.61 (EtOAc:Hex 2:3).

^1^H NMR (500.13 MHz, CDCl_3_, 25 °C) δ = 5.44 (dd, 1H, *J*_2,3_ = 10.2, *J*_3,4_ = 9.3 Hz, H-3), 4.88 (d, 1H, *J*_1,2_ = 3.6 Hz, H-1), 4.86 (dd, 1H, *J*_4,5_ = 9.9 Hz, H-4), 4.83 (dd, 1H, H-2), 3.89 (ddd, 1H, *J*_5,6a_ = 2.6, *J*_5,6b_ = 9.1 Hz, H-5), 3.41 (s, 3H, 1-OC**H**_**3**_), 2.99 (br s, 1H, cluster-C**H**), 2.89 (dd, 1H, *J*_6a,6b_ = −13.6 Hz, H-6a), 2.83 (dd, 1H, H-6b), 2.08 (s, 3H, 4-OCOC**H**_**3**_), 2.07 (s, 3H, 2-OCOC**H**_**3**_), 2.00 (s, 3H, 3-OCOC**H**_**3**_), and 3.15–1.54 (br m, 10H, cluster-B**H**) ppm.

^13^C{^1^H} NMR (125.76 MHz, CDCl_3_, 25 °C) δ = 170.3, 170.1, 170.0 (2-O**C**OCH_3_, 3-O**C**OCH_3_ and 4-O**C**OCH_3_), 96.6 (C-1), 74.2 (cluster-**C**), 71.9 (C-4), 70.9 (C-2), 69.9 (C-3), 68.2 (C-5), 55.7 (1-O**C**H_3_), 55.7 (cluster-**C**H), 37.8 (C-6), 20.9 and 20.8 (2-OCO**C**H_3_, 3-OCO**C**H_3_ and 4-OCO**C**H_3_) ppm.

^11^B{^1^H} NMR (160.46 MHz, CDCl_3_, 25 °C) δ = −3.6, −10.4, −13.3, and −14.5 ppm.

HRMS: *m*/*z* calcd. for B_10_C_15_H_30_NaO_8_S [M + Na]^+^: 503.2490, found: 503.2504.

##### 4.1.1.4 Methyl 2,3,4-Tri-*O*-acetyl-6-deoxy-6-thio-(1,2-dicarba-closo-dodecaboran-9-yl)-α-D-glucopyranoside (**8**)

Compound **5** (0.58 g, 1.34 mmol, 1.00 equiv) and 9-(mercapto)-1,2-dicarba-*closo-*dodecaborane(12) (0.26 g, 1.48 mmol, 1.10 equiv) were placed in an oven-dried Schlenk flask under an inert atmosphere. The mixture was suspended in dry acetone (25 mL), and K_2_CO_3_ (0.41 g, 2.95 mmol, 2.20 equiv) was added. The reaction mixture was allowed to stir overnight at 60 °C. After cooling to room temperature, 10 mL of saturated aqueous ammonium chloride solution was added and the pH was adjusted to 7 using 1 M HCl. Acetone was removed under reduced pressure, and the resulting aqueous solution was extracted with ethyl acetate (3 × 30 mL). The combined organic layers were washed with water and brine (30 mL) and dried over magnesium sulfate. The crude product was purified by column chromatography (EtOAc:Hex 1:4). Compound **8** was obtained as a colorless foam (0.51 g, 1.08 mmol, 80%). TLC: *R_f_*: 0.35 (EtOAc:Hex 2:3).

^1^H NMR (500.13 MHz, CDCl_3_, 25 °C) δ = 5.43 (dd, 1H, *J*_2,3_ = 10.2, *J*_3,4_ = 9.3 Hz, H-3), 4.91 (d, 1H, , *J*_1,2_ = 3.7 Hz, H-1), 4.87 (dd, 1H, H-2), 4.86 (dd, 1H, *J*_4,5_ = 10.0 Hz, H-4), 3.84 (ddd, 1H, *J*_5,6a_ = 2.4, *J*_5,6b_ = 9.4 Hz, H-5), 3.58 and 3.47 (each br s, each 1H, cluster-C**H**), 3.45 (s, 3H, 1-OC**H**_**3**_), 2.63 (dd, 1H, *J*_6a,6b_ = −13.6 Hz, H-6a), 2.56 (dd, 1H, H-6b), 2.07 (s, 3H, 2-OCOC**H**_**3**_), 2.04 (s, 3H, 4-OCOC**H**_**3**_), 2.00 (s, 3H, 3-OCOC**H**_**3**_), and 3.08–1.51 (br m, 9H, cluster-B**H**) ppm.

^13^C{^1^H} NMR (125.76 MHz, CDCl_3_, 25 °C) δ = 170.3, 170.3, 170.0 (2-O**C**OCH_3_, 3-O**C**OCH_3_ and 4-O**C**OCH_3_), 96.4 (C-1), 72.3 (C-4), 71.3 (C-2), 70.4 (C-3), 69.6 (C-5), 55.4 (1-O**C**H_3_), 53.0 and 47.8 (both cluster-**C**H), 33.7 (C-6), and 20.9 and 20.8 (2-OCO**C**H_3_, 3-OCO**C**H_3_ and 4-OCO**C**H_3_) ppm.

^11^B{^1^H} NMR (160.46 MHz, CDCl_3_, 25 °C) δ = 7.1, −2.5, −8.8, −14.5, and −15.5 ppm.

HRMS: *m*/*z* calcd. for B_10_C_15_H_30_NaO_8_S [M + Na]^+^: 503.2490, found: 503.2458.

##### 4.1.1.5 Methyl 2,3,4-Tri-*O*-acetyl-6-deoxy-6-thio-(1,7-dicarba-closo-dodecaboran-9-yl)-α-d-glucopyranoside (**9**)

Compound **5** (0.40 g, 0.93 mmol, 1.00 equiv) and 9-(mercapto)-1,7-dicarba-closo-dodecaborane(12) (0.25 g, 1.39 mmol, 1.50 equiv) were placed in an oven-dried Schlenk flask under an inert atmosphere. The mixture was suspended in dry acetone (25 mL), and DIPEA (0.47 mL, 2,79 mmol, 3,00 equiv) was added. The reaction was allowed to stir overnight at 60 °C. After cooling to room temperature, 10 mL of saturated aqueous ammonium chloride solution was added, and the pH was adjusted to 7 using 1 M HCl. Acetone was removed under reduced pressure, and the resulting aqueous solution was extracted with ethyl acetate (3 × 30 mL). The combined organic layers were washed with water and brine (30 mL) and dried over magnesium sulfate. The crude product was purified by column chromatography (EtOAc:Hex 1:1). Compound **9** was obtained as a colorless foam (0.40 g, 0.84 mmol, 90%). TLC: *R_f_*: 0.46 (EtOAc:Hex 2:3).

^1^H NMR (500.13 MHz, CDCl_3_, 25 °C) δ = 5.45 (dd, 1H, *J*_2,3_ = 10.3, *J*_3,4_ = 9.3 Hz, H-3), 4.94 (d, 1H, *J*_1,2_ = 3.7 Hz, H-1), 4.90 (dd, 1H, *J*_4,5_ = 10.0 Hz, H-4), 4.88 (dd, 1H, H-2), 3.90 (ddd, 1H, *J*_5,6a_ = 2.4, *J*_5,6b_ = 9.3 Hz, H-5), 3.47 (s, 3H, 1-OC**H**_**3**_), 2.97 (br s, 2H, cluster-C**H**), 2.73 (dd, 1H, *J*_6a,6b_ = −13.6 Hz, H-6a), 2.65 (dd, 1H, H-6b), 2.07 (s, 3H, 4-OCOC**H**_**3**_), 2.04 (s, 3H, 2-OCOC**H**_**3**_), 2.00 (s, 3H, 3-COC**H**_**3**_), and 3.15–1.54 (br m, 9H, cluster-B**H**) ppm.

^13^C{^1^H} NMR (125.76 MHz, CDCl_3_, 25 °C) δ = 170.3, 170.2, 170.0 (2-O**C**OCH_3_, 3-O**C**OCH_3_ and 4-O**C**OCH_3_), 96. 5 (C-1), 72.3 (C-4), 71.2 (s, C-2), 70.4 (s, C-3), 69.6 (s, C-5), 55.5 (1-O**C**H_3_), 54.2 (both cluster-**C**H), 34.1 (C-6), 20.9 and 20.8 (2-OCO**C**H_3_, 3-OCO**C**H_3_ and 4-OCO**C**H_3_) ppm.

^11^B{^1^H} NMR (160.46 MHz, CDCl_3_, 25 °C) δ = 0.2, −6.5, −10.0, −13.2, −13.9, −17.6, and −20.5 ppm.

HRMS: *m*/*z* calcd. for B_10_C_15_H_30_NaO_8_S [M + Na]^+^: 503.2490, found: 503.2466.

##### 4.1.1.6 6-Deoxy-6-thio-(1,2-dicarba-closo-dodecaboran-1-yl)-d-glucopyranose (**1**)

Compound **6** (0.043 g, 0.090 mmol) was dissolved in 2 M HCl (3 mL) and stirred at 90 → 115 °C. After 7 h, the reaction mixture was brought to room temperature, cooled with an ice bath, and neutralized by the addition of Na_2_CO_3_. The reaction mixture was concentrated, and the residue was dissolved in MeOH (10 mL) and stirred for 15 min, after which the formed solid was removed by filtration. The filtrate was concentrated, and the crude product was purified by column chromatography (DCM:MeOH 7:1). Compound **1** was obtained as a white solid (0.009 g, 0.026 mmol, 35%, α/β 56:44). TLC: *R_f_*: 0.34 (DCM:MeOH 7:1).

α anomer: ^1^H NMR (500.13 MHz, CD_3_OD, 25 °C): δ = 5.02 (d, 1H, *J*_1,2_ = 3.7 Hz, H-1), 4.66 (br s, 1H, cluster-C**H**), 3.92 (ddd, 1H, *J*_4,5_ = 9.7, *J*_5,6a_ = 2.9, *J*_5,6b_ = 8.2 Hz, H-5), 3.62 (dd, 1H, *J*_2,3_ = 9.6, *J*_3,4_ = 8.9 Hz, H-3), 3.44 (dd, 1H, *J*_6a,6b_ = −12.6 Hz, H-6a), 3.32 (dd, 1H, H-2), 3.14 (dd, 1H, H-4), 3.10 (dd, 1H, H-6b), and 3.05–1.50 (br m, 10H, cluster-B**H**) ppm.

^13^C{^1^H} NMR (125.76 MHz, CD_3_OD, 25 °C): δ = 94.0 (C-1), 77.0 (cluster-**C**), 74.8 (C-4), 74.6 (C-3), 73.7 (C-2), 71.2 (C-5), 70.1 (cluster-**C**H), and 40.4 (C-6) ppm.

β anomer: ^1^H NMR (500.13 MHz, CD_3_OD, 25 °C): δ = 4.73 (br s, 1H, cluster-C**H**), 4.43 (d, 1H, *J*_1,2_ = 7.8 Hz, H-1), 3.46 (dd, 1H, *J*_5,6a_ = 2.8, *J*_6a,6b_ = −13.2 Hz, H-6a), 3.40 (ddd, 1H, *J*_4,5_ = 9.5, *J*_5,6b_ = 8.2 Hz, H-5), 3.30 (dd, 1H, *J*_2,3_ = 9.4, *J*_3,4_ = 8.9 Hz, H-3), 3.17 (dd, 1H, H-4), 3.11 (dd, 1H, H-6b), 3.10 (dd, 1H, H-2), and 3.05–1.50 (br m, 10H, cluster-B**H**) ppm.

^13^C{^1^H} NMR (125.76 MHz, CD_3_OD, 25 °C): δ = 98.3 (C-1), 77.7 (C-3), 76.8 (cluster-**C**), 76.2 (C-2), 76.0 (C-5), 74.4 (C-4), 69.9 (cluster-**C**H), and 40.1 (C-6) ppm.

^11^B{^1^H} NMR (160.46 MHz, CD_3_OD, 25 °C): δ = −1.1, −4.5, −8.5, and −11.7 ppm.

HRMS *m*/*z*: calcd for C_8_H_22_B_10_NaO_5_S [M + Na]^+^, 363.2016; found, 363.2002.

##### 4.1.1.7 6-Deoxy-6-thio-(1,7-dicarba-closo-dodecaboran-1-yl)-d-glucopyranose (**2**)

Compound **7** (0.045 g, 0.094 mmol) was dissolved in 4 M HCl (3 mL) and stirred at 90 → 110 °C. After 4 h, the reaction mixture was brought to room temperature, then cooled with an ice bath, and neutralized by the addition of Na_2_CO_3_. The reaction mixture was concentrated, and the residue was dissolved in MeOH (10 mL) and stirred for 15 min, after which the formed solid was removed by filtration. The filtrate was concentrated, and the crude product was purified by column chromatography (DCM:MeOH 7:1). Compound **2** was obtained as a white solid (0.014 g, 0.040 mmol, 43%, α/β 49:51). TLC: *R_f_*: 0.33 (DCM:MeOH 7:1).

α anomer: ^1^H NMR (500.13 MHz, CD_3_OD, 25 °C): δ = 5.01 (d, 1H, *J*_1,2_ = 3.7 Hz, H-1), 3.89 (ddd, 1H, *J*_4,5_ = 9.7, *J*_5,6a_ = 2.5, *J*_5,6b_ = 8.6 Hz, H-5), 3.61 (dd, 1H, *J*_2,3_ = 9.6, *J*_3,4_ = 9.0 Hz, H-3), 3.60 (br s, 1H, cluster-C**H**), 3.31 (dd, 1H, H-2), 3.29 (dd, 1H, *J*_6a,6b_ = −12.4 Hz, H-6a), 3.10 (dd, 1H, H-4), 2.89 (dd, 1H, H-6b), and 2.83–1.48 (br m, 10H, cluster-B**H**) ppm.

^13^C{^1^H} NMR (125.76 MHz, CD_3_OD, 25 °C): δ = 93.9 (C-1), 75.0 (C-4), 74.6 (C-3), 73.8 (C-2), 73.0 (cluster-**C**), 71.1 (C-5), 57.6 (cluster-**C**H), and 39.8 (C-6) ppm.

β anomer: ^1^H NMR (500.13 MHz, CD_3_OD, 25 °C): δ = 4.41 (d, 1H, *J*_1,2_ = 7.9 Hz, H-1), 3.60 (br s, 1H, cluster-C**H**), 3.35 (ddd, 1H, *J*_4,5_ = 9.7, *J*_5,6a_ = 2.3, *J*_5,6b_ = 8.7 Hz, H-5), 3.32 (dd, 1H, *J*_6a,6b_ = −12.7 Hz, H-6a), 3.29 (dd, 1H, *J*_2,3_ = 9.5, *J*_3,4_ = 8.6 Hz, H-3), 3.13 (dd, 1H, H-4), 3.09 (dd, 1H, H-2), 2.91 (dd, 1H, H-6b), and 2.83–1.48 (br m, 10H, cluster-B**H**) ppm.

^13^C{^1^H} NMR (125.76 MHz, CD_3_OD, 25 °C): δ = 98.3 (C-1), 77.7 (C-3), 76.2 (C-2), 76.1 (C-5), 74.7 (C-4), 73.0 (cluster-**C**), 57.6 (cluster-**C**H), and 39.6 (C-6) ppm.

^11^B{^1^H} NMR (160.46 MHz, CD_3_OD, 25 °C): δ = −2.8, −9.4, −10.1, −12.7, and −13.7 ppm.

HRMS *m*/*z*: calcd for C_8_H_22_B_10_NaO_5_S [M + Na]^+^, 363.2016; found, 363.1998.

##### 4.1.1.8 6-Deoxy-6-thio-(1,2-dicarba-closo-dodecaboran-9-yl)-d-glucopyranose (**3**)

Compound **8** (0.089 g, 0.185 mmol) was dissolved in 1 M HCl (5 mL) and stirred at 90 → 120 °C. After 7 h, the reaction mixture was brought to room temperature, then cooled with an ice bath, and neutralized by the addition of Na_2_CO_3_. The reaction mixture was concentrated, and the residue was dissolved in MeOH (15 mL) and stirred for 15 min, after which the formed solid was removed by filtration. The filtrate was concentrated, and the crude product was purified by column chromatography (DCM:MeOH 7:1). Compound **3** was obtained as a white solid (0.047 g, 0.138 mmol, 75%, α/β 48:52). TLC: *R_f_*: 0.30 (DCM:MeOH 7:1).

α anomer: ^1^H NMR (500.13 MHz, CD_3_OD, 25 °C): δ = 5.04 (d, 1H, *J*_1,2_ = 3.8 Hz, H-1), 4.29 (br s, 2H, cluster-C**H**), 3.83 (ddd, 1H, *J*_4,5_ = 9.7, *J*_5,6a_ = 2.7, *J*_5,6b_ = 8.2 Hz, H-5), 3.61 (dd, 1H, *J*_2,3_ = 9.6, *J*_3,4_ = 8.9 Hz, H-3), 3.32 (dd, 1H, H-2), 3.13 (dd, 1H, H-4), 2.97 (dd, 1H, *J*_6a,6b_ = −12.8 Hz, H-6a), 2.57 (dd, 1H, H-6b), and 2.94–1.46 (br m, 9H, cluster-B**H**) ppm.

^13^C{^1^H} NMR (125.76 MHz, CD_3_OD, 25 °C): δ = 93.8 (C-1), 74.9 (C-4), 74.7 (C-3), 73.8 (C-2), 72.5 (C-5), 55.7 and 50.0 (both cluster-**C**H), and 35.5 (C-6) ppm.

β anomer: ^1^H NMR (500.13 MHz, CD_3_OD, 25 °C): δ = 4.42 (d, 1H, *J*_1,2_ = 7.8 Hz, H-1), 4.37 (br s, 2H, cluster-C**H**), 3.29 (dd, 1H, *J*_2,3_ = 9.4, *J*_3,4_ = 9.3 Hz, H-3), 3.27 (ddd, 1H, *J*_4,5_ = 9.3, *J*_5,6a_ = 2.4, *J*_5,6b_ = 8.8 Hz, H-5), 3.12 (dd, 1H, H-4), 3.10 (dd, 1H, H-2), 3.01 (dd, 1H, *J*_6a,6b_ = −13.2 Hz, H-6a), 2.53 (dd, 1H, H-6b), and 2.94–1.46 (br m, 9H, cluster-B**H**) ppm.

^13^C{^1^H} NMR (125.76 MHz, CD_3_OD, 25 °C): δ = 98.1 (C-1), 77.9 (C-3), 77.8 (C-5), 76.3 (C-2), 75.1 (C-4), 55.7 and 50.0 (both cluster-**C**H), and 35.3 (C-6) ppm.

^11^B{^1^H} NMR (160.46 MHz, CD_3_OD, 25 °C): δ = 7.4, −2.3, −8.3, −13.2, −13.9, and −14.6 ppm.

HRMS *m*/*z*: calcd for C_8_H_22_B_10_NaO_5_S [M + Na]^+^, 363.2016; found, 363.2035.

##### 4.1.1.9 6-Deoxy-6-thio-(1,7-dicarba-closo-dodecaboran-9-yl)-d-glucopyranose (**4**)

Compound **9** (0.099 g, 0.206 mmol) was dissolved in 1 M HCl (6 mL) and stirred at 80 → 115 °C. After 7 h, the reaction mixture was brought to room temperature, cooled with an ice bath, and neutralized by the addition of Na_2_CO_3_. The reaction mixture was concentrated, and the residue was dissolved in MeOH (15 mL) and stirred for 15 min, after which the formed solid was removed by filtration. The filtrate was concentrated, and the crude product was purified by column chromatography (DCM:MeOH 7:1). Compound **4** was obtained as a white solid (0.052 g, 0.153 mmol, 74%, α/β 55:45). TLC: *R_f_*: 0.32 (DCM:MeOH 7:1).

α anomer: ^1^H NMR (500.13 MHz, CD_3_OD, 25 °C): δ = 5.07 (d, 1H, *J*_1,2_ = 3.8 Hz, H-1), 3.89 (ddd, 1H, *J*_4,5_ = 9.7, *J*_5,6a_ = 2.7, *J*_5,6b_ = 8.2 Hz, H-5), 3.63 (dd, 1H, *J*_2,3_ = 9.6, *J*_3,4_ = 8.9 Hz, H-3), 3.55 (br s, 2H, cluster-C**H**), 3.35 (dd, 1H, H-2), 3.17 (dd, 1H, H-4), 3.08 (dd, 1H, *J*_6a,6b_ = −12.7 Hz, H-6a), 2.68 (dd, 1H, H-6b), and 3.04–1.50 (br m, 9H, cluster-B**H**) ppm.

^13^C{^1^H} NMR (125.76 MHz, CD_3_OD, 25 °C): δ = 93.8 (C-1), 75.0 (C-4), 74.7 (C-3), 73.8 (C-2), 72.5 (C-5), 56.0 (both cluster-**C**H), and 35.9 (C-6) ppm.

β anomer: ^1^H NMR (500.13 MHz, CD_3_OD, 25 °C): δ = 4.46 (d, 1H, *J*_1,2_ = 7.8 Hz, H-1), 3.55 (br s, 2H, cluster-C**H**), 3.33 (ddd, 1H, *J*_4,5_ = 9.0, *J*_5,6a_ = 2.6, *J*_5,6b_ = 9.8 Hz, H-5), 3.31 (dd, 1H, *J*_2,3_ = 9.2, *J*_3,4_ = 9.4 Hz, H-3), 3.16 (dd, 1H, H-4), 3.13 (dd, 1H, H-2), 3.12 (dd, 1H, *J*_6a,6b_ = −12.9 Hz, H-6a), 2.65 (dd, 1H, H-6b), and 3.04–1.50 (br m, 9H, cluster-B**H**) ppm.

^13^C{^1^H} NMR (125.76 MHz, CD_3_OD, 25 °C): δ = 98.2 (C-1), 77.9 (C-3), 77.8 (C-5), 76.3 (C-2), 74.9 (C-4), 56.0 (both cluster-**C**H), and 35.7 (C-6) ppm.

^11^B{^1^H} NMR (160.46 MHz, CD_3_OD, 25 °C): δ = 1.0, −6.1, −9.4, −12.4, −13.4, −16.8, and −19.8 ppm.

HRMS *m*/*z*: calcd for B_10_C_8_H_22_NaO_5_S [M + Na]^+^, 363.2016; found, 363.1976.

##### 4.1.1.10 Methyl 6-Deoxy-6-thio-(1,7-dicarba-closo-dodecaboran-1-yl)-d-glucopyranoside (**10**)

Compound **7** (0.049 g, 0.105 mmol) was dissolved in 1 M HCl (3 mL) and stirred at 105 °C. After 2 h, the reaction mixture was brought to room temperature and neutralized with the addition of Na_2_CO_3_ at 0 °C. The reaction mixture was concentrated, and the residue was dissolved in MeOH (10 mL) and stirred for 15 min, after which the formed solid was removed by filtration. The filtrate was concentrated, and the crude product was purified by column chromatography (DCM:MeOH 7:1). Compound **10** was obtained as a clear oil (0.036 g, 0.088 mmol, 84%). TLC: *R_f_*: 0.68 (DCM/MeOH 7:1).

^1^H NMR (500.13 MHz, CD_3_OD, 25 °C): δ = 4.59 (d, 1H, *J*_1,2_ = 3.8 Hz, H-1), 3.63 (br s, 1H, cluster-C**H**), 3.59 (ddd, 1H, *J*_4,5_ = 9.7, *J*_5,6a_ = 2.4, *J*_5,6b_ = 9.4 Hz, H-5), 3.55 (dd, 1H, *J*_2,3_ = 9.7, *J*_3,4_ = 8.9 Hz, H-3), 3.38 (s, 3H, 1-OC**H**_**3**_), 3.36 (dd, 1H, H-2), 3.31 (dd, 1H, *J*_6a,6b_ = −12.9 Hz, H-6a), 3.09 (dd, 1H, H-4), 2.87 (dd, 1H, H-6b), and 2.81–1.48 (br m, 10H, cluster-B**H**) ppm.

^13^C{^1^H} NMR (125.76 MHz, CD_3_OD, 25 °C): δ = 101.2 (C-1), 75.0 (C-4), 74.9 (C-3), 74.2 (cluster-**C**), 73.5 (C-2), 71.8 (C-5), 57.6 (cluster-**C**H), 55.7 (1-O**C**H_3_), and 39.6 (C-6) ppm.

^11^B{^1^H} NMR (160.46 MHz, CD_3_OD, 25 °C): δ = −2.2, −8.8, −9.5, −12.1, and −13.1 ppm.

HRMS *m*/*z*: calcd for B_10_C_9_H_24_NaO_5_S [M + Na]^+^, 377.2173; found, 377.2105.

##### 4.1.1.11 Methyl 6-Deoxy-6-thio-(1,7-dicarba-closo-dodecaboran-9-yl)-d-glucopyranoside (**11**)

Compound **9** (0.052 g, 0.112 mmol) was dissolved in 1 M HCl (5 mL) and stirred at 85 °C. After 4 h, the reaction mixture was brought to room temperature and neutralized with the addition of Na_2_CO_3_ at 0 °C. The reaction mixture was concentrated, and the residue was dissolved in MeOH (10 mL) and stirred for 15 min, after which the formed solid was removed by filtration. The filtrate was concentrated, and the crude product was purified by column chromatography (DCM:MeOH 7:1). Compound **11** was obtained as a clear oil (0.039 g, 0.083 mmol, 74%). TLC: *R_f_*: 0.41 (DCM/MeOH 9:1).

^1^H NMR (500.13 MHz, CD_3_OD, 25 °C): δ = 4.63 (d, 1H, *J*_1,2_ = 3.8 Hz, H-1), 3.60 (ddd, 1H, *J*_4,5_ = 9.7, *J*_5,6a_ = 2.0, *J*_5,6b_ = 9.1 Hz, H-5), 3.57 (dd, 1H, *J*_2,3_ = 9.7, *J*_3,4_ = 8.9 Hz, H-3), 3.56 (br s, 2H, cluster-C**H**), 3.44 (s, 3H, 1-OC**H**_**3**_), 3.39 (dd, 1H, H-2), 3.11 (dd, 1H, H-4), 3.10 (dd, 1H, *J*_6a,6b_ = −13.2 Hz, H-6a), 2.60 (dd, 1H, H-6b), and 3.05–1.46 (br m, 9H, cluster-B**H**) ppm.

^13^C{^1^H} NMR (125.76 MHz, CD_3_OD, 25 °C): δ = 100.9 (C-1), 75.2 (C-4), 75.1 (C-3), 73.7 (C-2), 73.3 (C-5), 56.1 (both cluster-**C**H), 55.6 (1-O**C**H_3_), and 35.7 (C-6) ppm.

^11^B{^1^H} NMR (160.46 MHz, CD_3_OD, 25 °C): δ = 1.1, −6.0, −9.4, −12.4, −13.3, −16.7, and −19.7 ppm.

HRMS *m*/*z*: calcd for B_10_C_9_H_24_NaO_5_S [M + Na]^+^, 377.2173; found, 377.2139.

### 4.2 *In Vitro* Assessment Protocols

The CAL 27 (ATCC CRL-2095) cells used in all assays were acquired from ATCC (Manassas, VA, USA) and cultured in Dulbecco’s Modified Eagle Medium (DMEM) supplemented with l-glutamine (2.0 mM), heat-inactivated fetal bovine serum (10%), and penicillin (50 U/mL)-streptomycin (50 μg/mL) at 37 °C with 5% CO_2_ and 95% relative humidity.

#### 4.2.1 Affinity Studies

The GLUT1 affinity assay was performed according to our previously developed protocol.^13,14^ The cells were incubated at room temperature for 5 min with **1**–**4**, BSH, and BPA across the 0.1–1800 μM concentration range. In addition, the solutions contained 1.8 μM (0.1 mCi/mL) of [^14^C]-d-glucose in glucose-free HBSS (Hanks’ balanced salt solution) (250 μL). Ice-cold buffer was used to quench the reactions. Afterward, the cells were washed twice. Lysis was carried out with 0.1 M NaOH (250 μL) before further mixing with emulsifier safe cocktail (1.0 mL) (PerkinElmer, Waltham, MA, USA). Liquid scintillation counter (MicroBeta^2^ counter, PerkinElmer, Waltham, MA, USA) was used to assess the radioactivity, and the IC_50_ values were determined by nonlinear regression analysis.

#### 4.2.2 Cellular Uptake Studies

The cells were first pre-incubated. The substrates were then added in different concentration (10–400 μM in 250 μL of HBSS) and incubation was continued for 5, 30, and 120 min at room temperature. Ice-cold buffer was used to quench the reaction at the set time points. Washing and lysis was carried out as mentioned above. A combined sample was prepared in an Eppendorf tube by addition of cell lysate from four wells. The combined sample was centrifuged at +4 °C. From the supernatant of every sample, 800 μL was taken and digested in 1.0 mL of HNO_3_ (TraceMetal grade, Fisher Chemical) for 24 h. Milli-Q water (USF Elga Purelab Ultra) was added until the sample volume was 10 mL, and the boron contents were assessed in triplicates by ICP-MS. More details on the ICP method can be found in our earlier work.^13,14^ PerkinElmer Syngistix Data Analysis Software was used for data analysis and GraphPad Prism v.5.03 software (GraphPad Software, San Diego, Ca, USA) for statistical analysis.

#### 4.2.3 Cytotoxicity Studies

The CAL 27 cells were seeded on an opaque-walled 96-well plate at a density of 5000 cells per well and incubated overnight. The cell culture media containing different substrates at concentrations of 5, 25, 50, 125, and 250 μM were incubated with the cells for 6 and 24 h at 37 °C, 5% CO_2_ atmosphere, and 95% relative humidity. At designated time points, the test media were disposed followed by washing of the cells two times with 1 × DPBS (pH 7.4). For the cell viability assay, 1× HBSS and CellTiter-Glo reagent (50 μL each) were added to each well and left for cell lysis at ambient temperature in the dark. The Synergy H1 Hybrid multimode microplate reader (BioTek, Winooski, VT, USA) was used to measure the luminescence signal of viable cells. After luminescent reading, the Pierce colorimetric bicinchoninic acid (BCA) protein assay (Thermo Fisher Scientific, Waltham, MA, USA) was used to quantify the total protein content of every sample. Briefly, 25 μL of the sample in each well was taken to a new transparent 96-well plate. Then, 200 μL of BCA working reagent was added and the plates were wrapped with aluminum foil and incubated at 37 °C for 30 min. The absorbance was recorded at 562 nm using the microplate reader. The protein content in each sample was calculated using the BSA standard curve (concentration range: 0–2000 μg/mL). The protein content was used for the normalization of the cell viability data. All assays were carried out in triplicate.

### 4.3 Computational and Docking Studies

Starting geometries for the studied molecules were built and these were first optimized with xtb (version 6.3.2) followed by conformational sampling with CREST (Conformer-Rotamer Ensemble Sampling Tool) using the GFN2-xTB method.^47^ The restrained electrostatic potential (RESP) partial charges were calculated for the CREST best conformation of each molecule.^48^ Since the conformation of the molecule affects the calculation of the partial charges, we wanted to first perform a small docking protocol to get a conformation that represents docked/bound structures well. A docking study was conducted, with 300 independent search runs. From this, the cluster with the lowest energy featuring the majority of structures was chosen as the representing one, and the lowest energy conformer was chosen for further use. Since AutoDock^49,50^ merges polar hydrogens for the docking, those hydrogens were added back to the ligand molecules in the next step of the geometry optimization. The RESP partial charge calculation was conducted for these, and the final results were obtained from a docking study featuring 2000 independent search runs for each ligand. A local minimum was identified on DFT level by optimizing the geometries with dispersion-corrected hybrid Tao–Perdew–Scuseria–Staroverov functional TPSSh-D3(BJ).^51−53^ The def2-TZVPP basis set was used.^54^ To compute the partial charges of the atoms, the RESP protocol was used.^48^ For RESP charge calculations, each ligand was split into two parts (one part consisting of the carborane, the sulfur atom, and the 6th position of the glucose backbone, and the other part comprising the carbohydrate core). Hydrogen atoms at the same carbon atom were treated as equal. Turbomole 7.4.1 was used for the optimization of the geometries,^55,56^ and NWChem 6.8.1 was used for RESP calculations.^57^

AutoDock 4.2.6 was used in molecular docking studies.^49,50^ In the carborane part, all rotatable bonds were rendered inactive, i.e., set as nonrotatable. Further, the torsional degrees of freedom for the carborane were set to 7 (torsdof 7). The XylE inward open 4QIQ^41^ and outward-open 6N3I^42^ PDB structures were used as the base. PyMOL was used to mutate these structures by replacing Gln-415 with Asn-415. The rotamer, in which the lowest number of clashes between neighboring amino acids was reported, was selected. The preparation of the transporter was performed in the following way: the ligand and small molecules present (Zn for 4QIQ) were removed, hydrogens were added and then merged, and Gasteiger partial charges were calculated. A grid size of 46 × 56 × 60 was selected with a 0.375 value for grid spacing. For the binding site to be covered by the grid box, the midpoint of the protein cavity was set as the center of the grid. The protein was kept in a rigid state during the docking studies while the torsional angles in the ligand were altered. Two separate dockings were performed for each ligand: the initial one with 300 independent search runs and the second one with 2000. The following parameters were employed: a maximum of 2.5 million energy evaluations and a maximum of 27,000 generations with a population size of 150. Further, the Lamarckian genetic algorithm (LGA) was used in the default setting, i.e., employing a 0.02 mutation rate and a 0.8 crossover rate, with the top individual moving onto the next generation. Last, ranking/clustering of conformations was done with a cluster RMS 2.0 Å.

The missing boron parameters in AutoDock were added to the parameter file as follows: R 2.285, Rii 4.57, epsilon 0.179, vol 49.9744. Remaining parameters were given analogous values as for carbon. The chosen parameters are based on the work of Oda *et al*.,^58^ as reproduced by Couto *et al*.^59^ The parameter set employed was thus atom_par B 4.57 0.179 49.9744–0.00143 0.0 0.0 0 −1 −1 0 # Boron for Carborane.

## Supporting Information Available

All NMR spectra (^1^H, ^13^C, and ^11^B) of synthesized compounds as well as the overall mean binding energy (MBE) of each glucoconjugate (PDF)
