# Supplementary material for: Synthesis and In Vitro Evaluation of a Set of 6-Deoxy-6-thio-carboranyl d-Glucoconjugates Shed Light on the Substrate Specificity of the GLUT1 Transporter
Source: ACS Omega. 2022 Aug 17;7(34):30376–88. doi: 10.1021/acsomega.2c03646 (PMC9434784; doi:10.1021/acsomega.2c03646)
Supplement: Supplementary file 1 — ao2c03646_si_001.pdf [file ao2c03646_si_001.pdf]

SUPPORTING INFORMATION

Synthesis and *In Vitro* Evaluation of a Set of 6-Deoxy-6-thio-Carboranyl D-Glucoconjugates Sheds Light on the Substrate Specificity of the GLUT1 Transporter

Jelena Matović,[1,‡] Juulia Järvinen,[2,‡] Iris K. Sokka,[1,‡] Philipp Stockmann,[3] Martin Kellert,[3] Surachet Imlimthan,[1] Mirkka Sarparanta,[1] Mikael P. Johansson,[1,4,5] Evamarie Hey-Hawkins,[3] Jarkko Rautio[2] and Filip S. Ekholm[1*]

[1] Department of Chemistry, University of Helsinki, Finland, P.O. Box 55, FI-00014 Helsinki, Finland

[2] School of Pharmacy, University of Eastern Finland, P.O. Box 1627, FI-70211 Kuopio, Finland

[3] Institute of Inorganic Chemistry, Leipzig University, D-04103 Leipzig, Germany

[4] Helsinki Institute of Sustainability Science, HELSUS, FI-00014 Helsinki, Finland

[5] CSC – IT Center for Science Ltd., P.O. Box 405, FI-02101 Espoo, Finland

[‡]Equal contributions

Contact: filip.ekholm@helsinki.fi

Table of contents

1. NMR Spectra of Synthesized Compounds........ 3
2. Molecular modeling........ 19

1. NMR Spectra of Synthesized Compounds

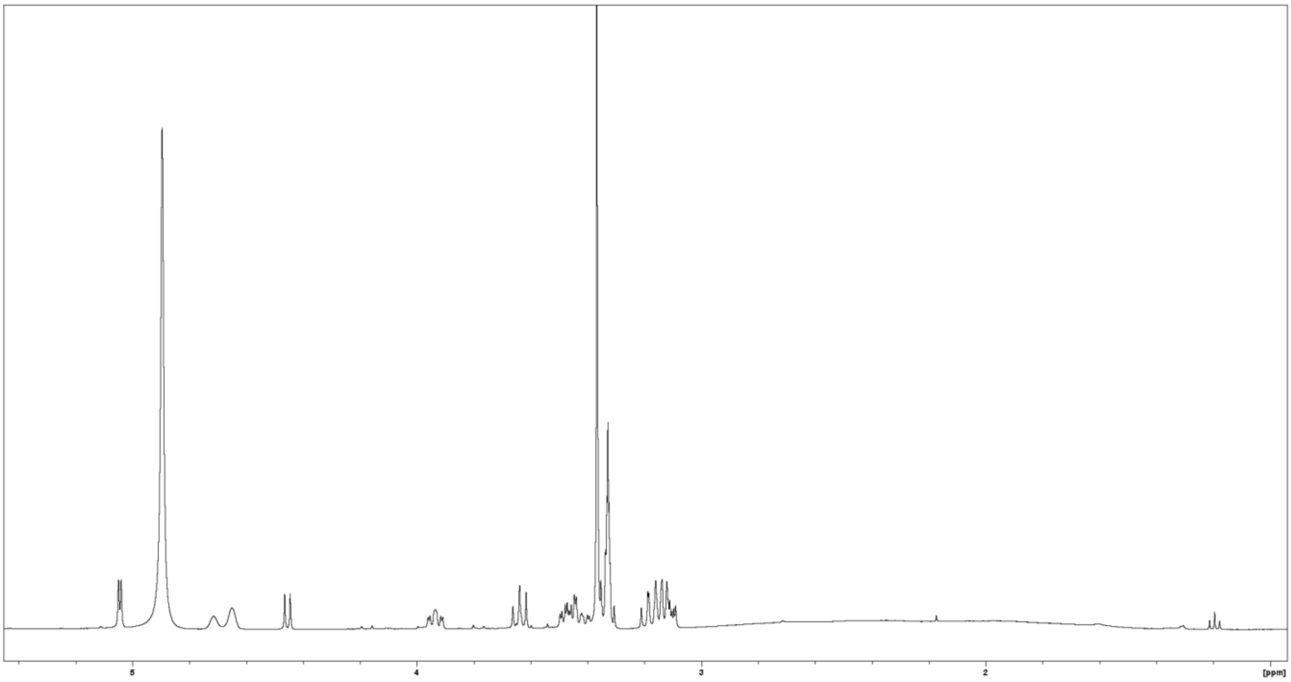

Figure S1. ^{1}H NMR spectrum of **1** (500.13 MHz, 25 °C, CD_3OD).

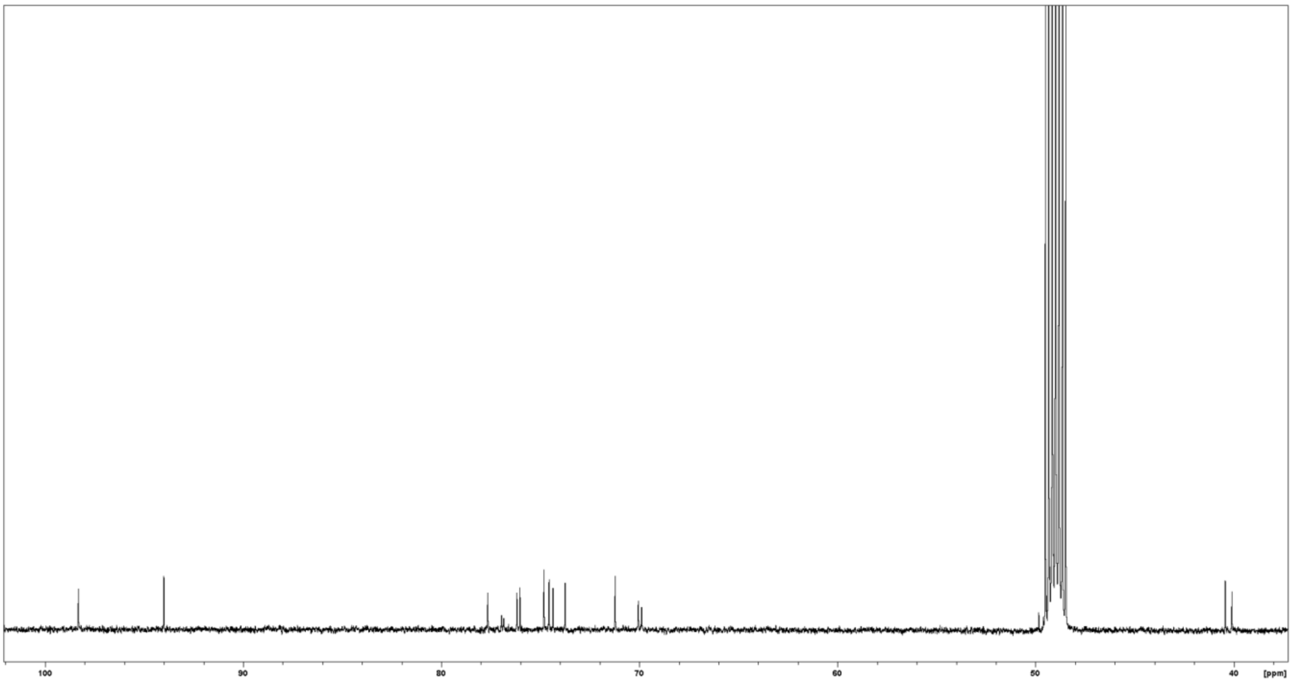

Figure S2. ^{13}C NMR spectrum of **1** (125.76 MHz, 25 °C, CD_3OD).

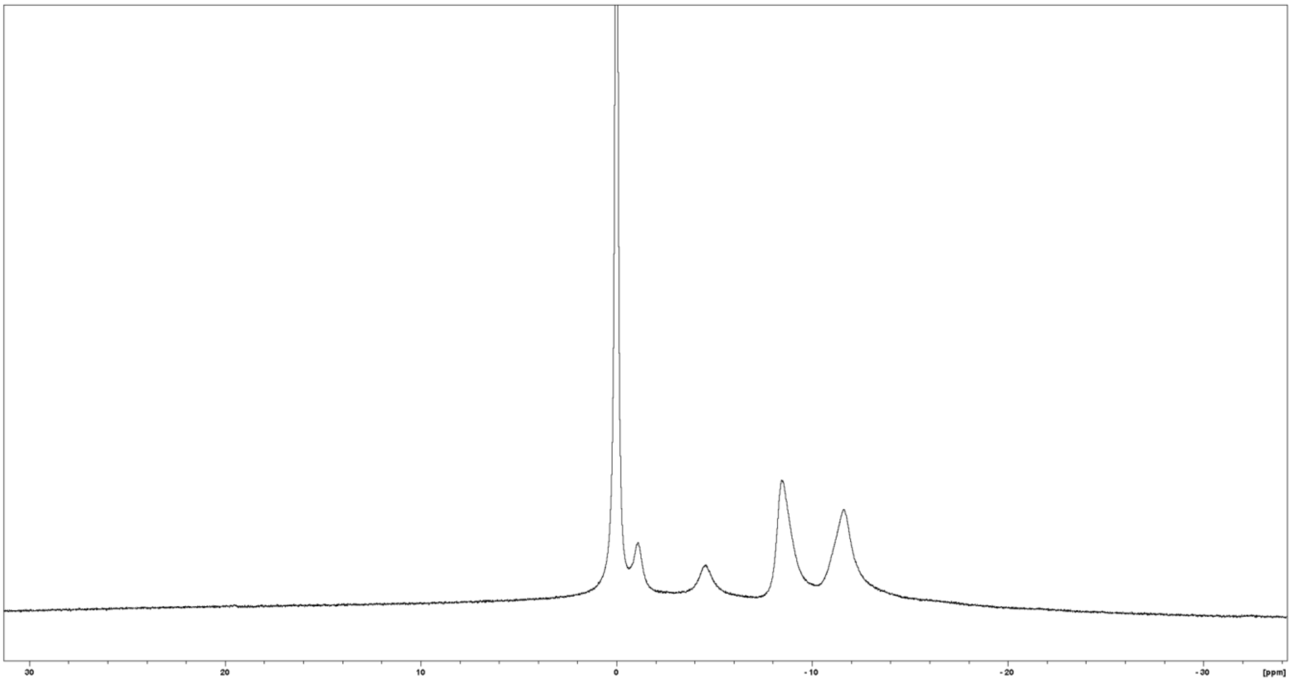

Figure S3. ^{11}B NMR spectrum of **1** (160.46 MHz, 25 °C, CD_3OD).

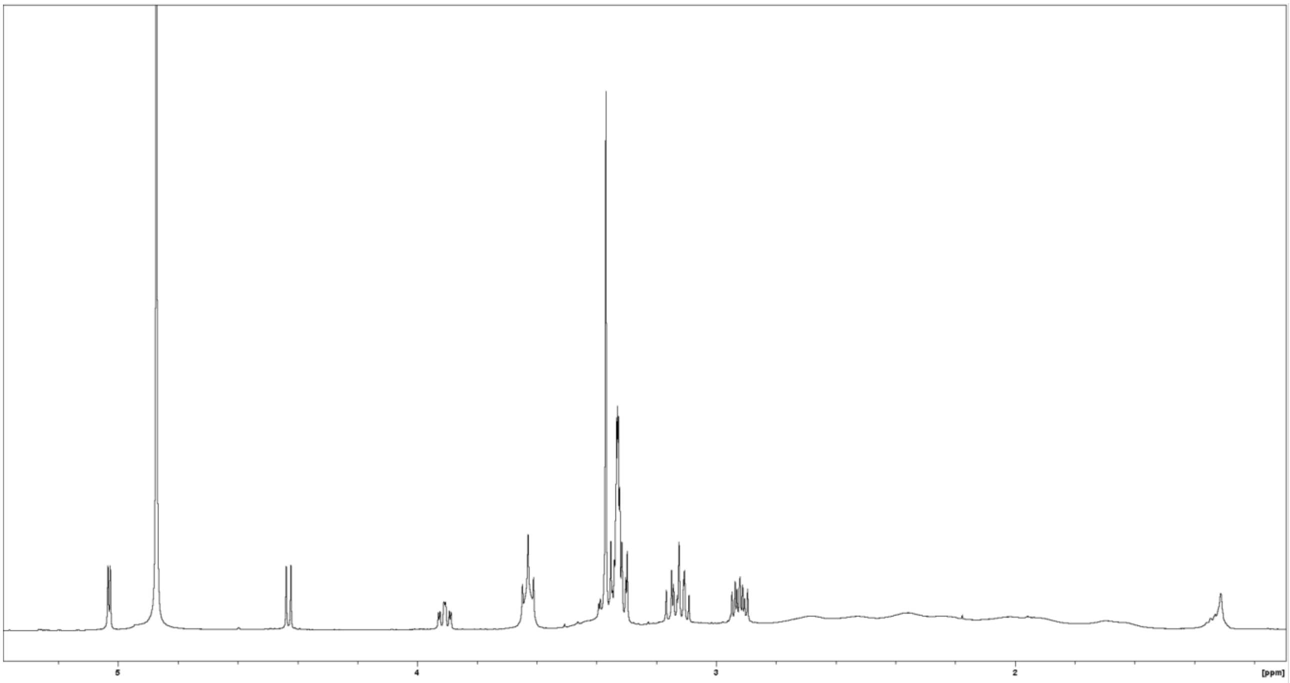

Figure S4. ^{1}H NMR spectrum of **2** (500.13 MHz, 25 °C, CD_3OD).

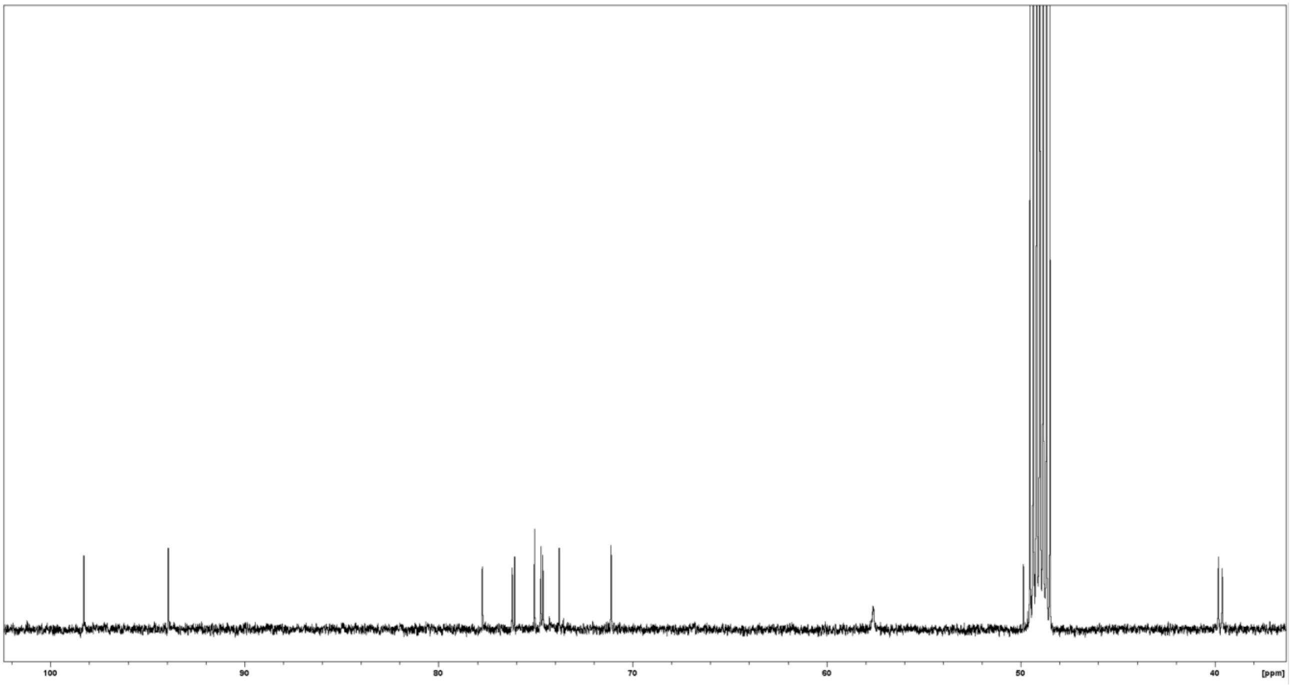

Figure S5. ^{13}C NMR spectrum of **2** (125.76 MHz, 25 °C, CD_3OD).

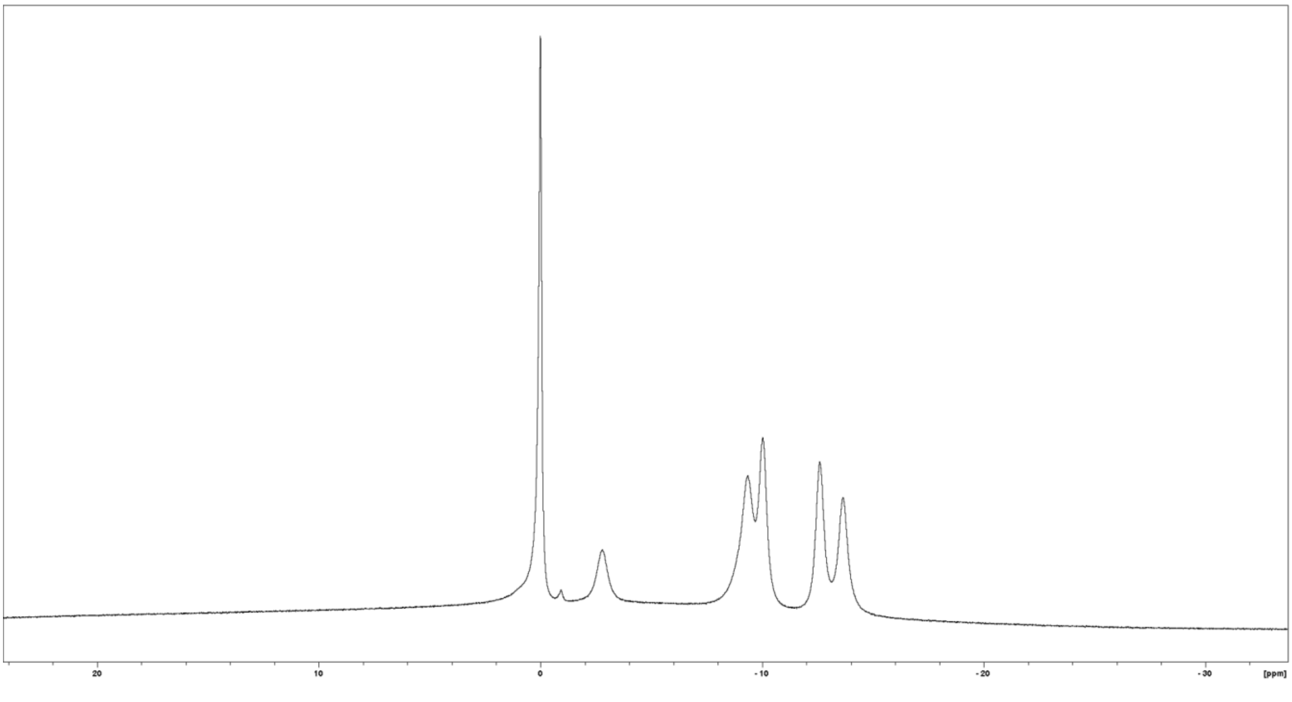

Figure S6. ^{11}B NMR spectrum of **2** (160.46 MHz, 25 °C, CD_3OD).

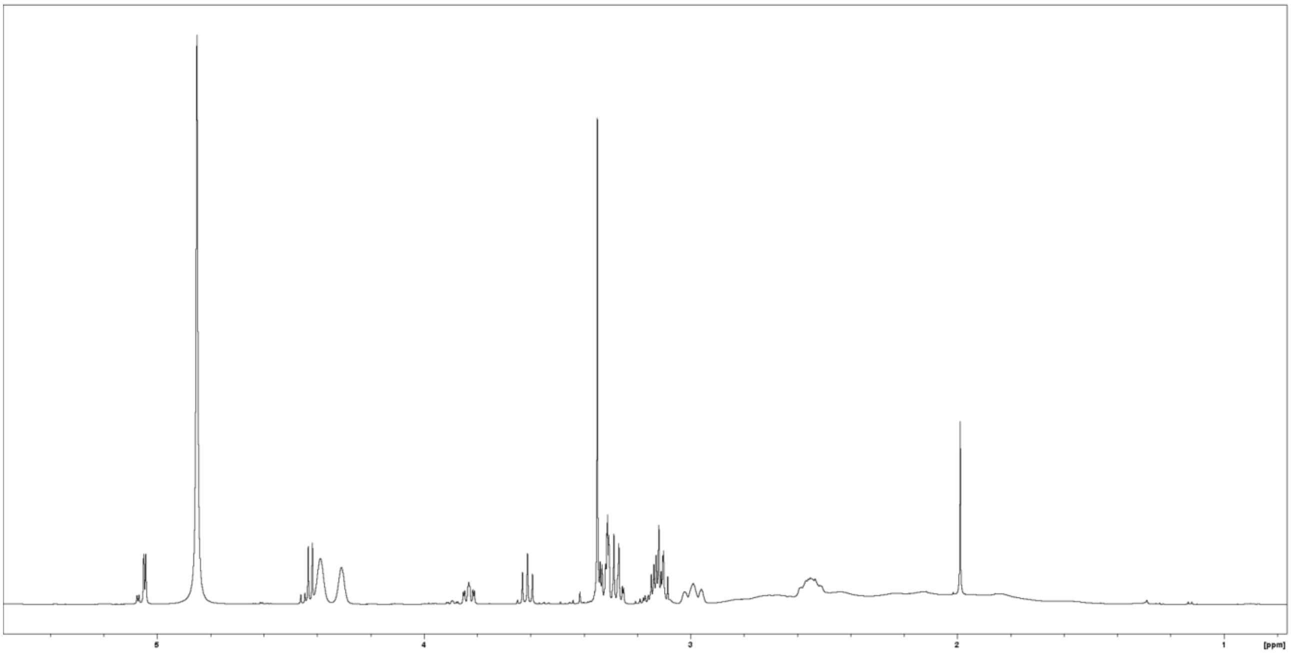

Figure S7. ^{1}H NMR spectrum of **3** (500.13 MHz, 25 °C, CD_3OD).

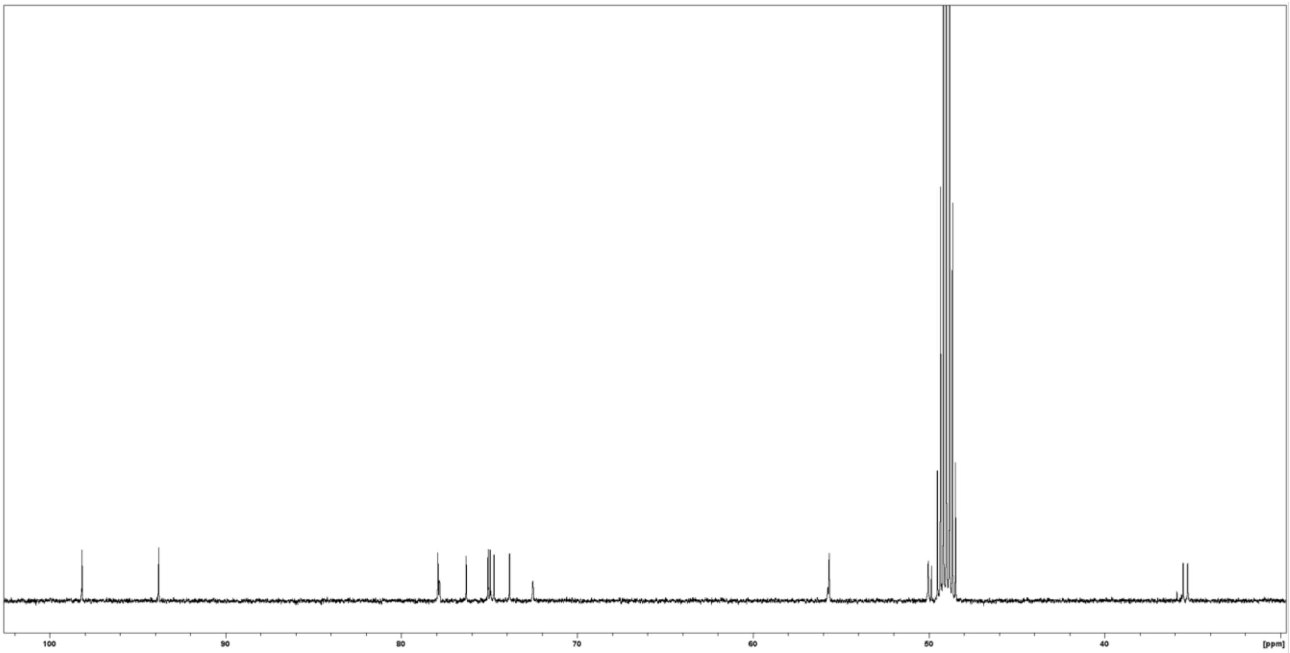

Figure S8. ^{13}C NMR spectrum of **3** (125.76 MHz, 25 °C, CD_3OD).

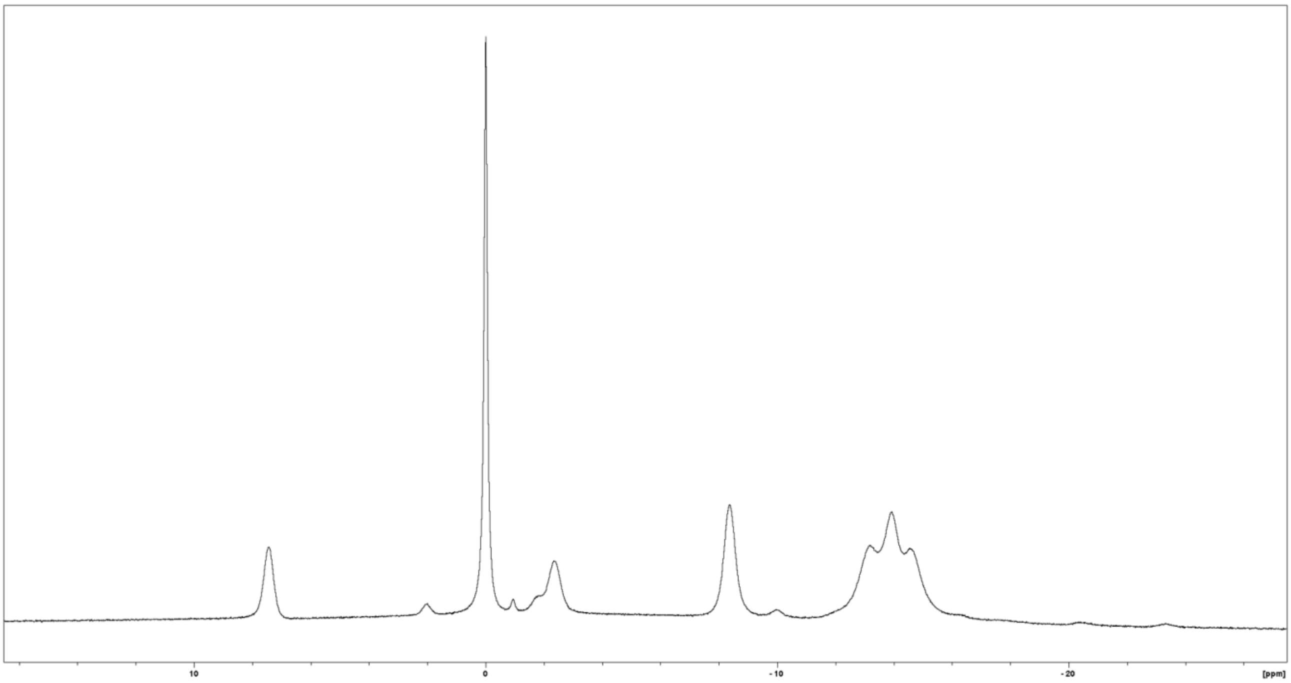

Figure S9. ^{11}B NMR spectrum of **3** (160.46 MHz, 25 °C, CD_3OD).

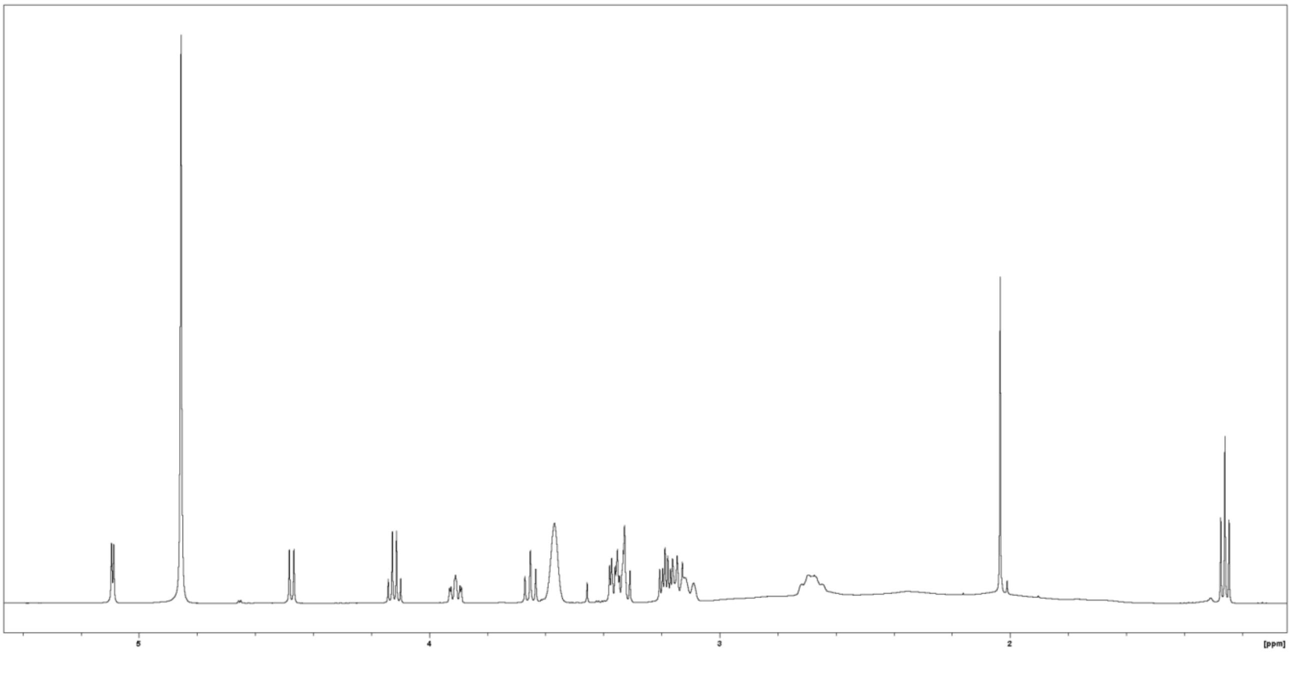

Figure S10. ^{1}H NMR spectrum of **4** (500.13 MHz, 25 °C, CD_3OD).

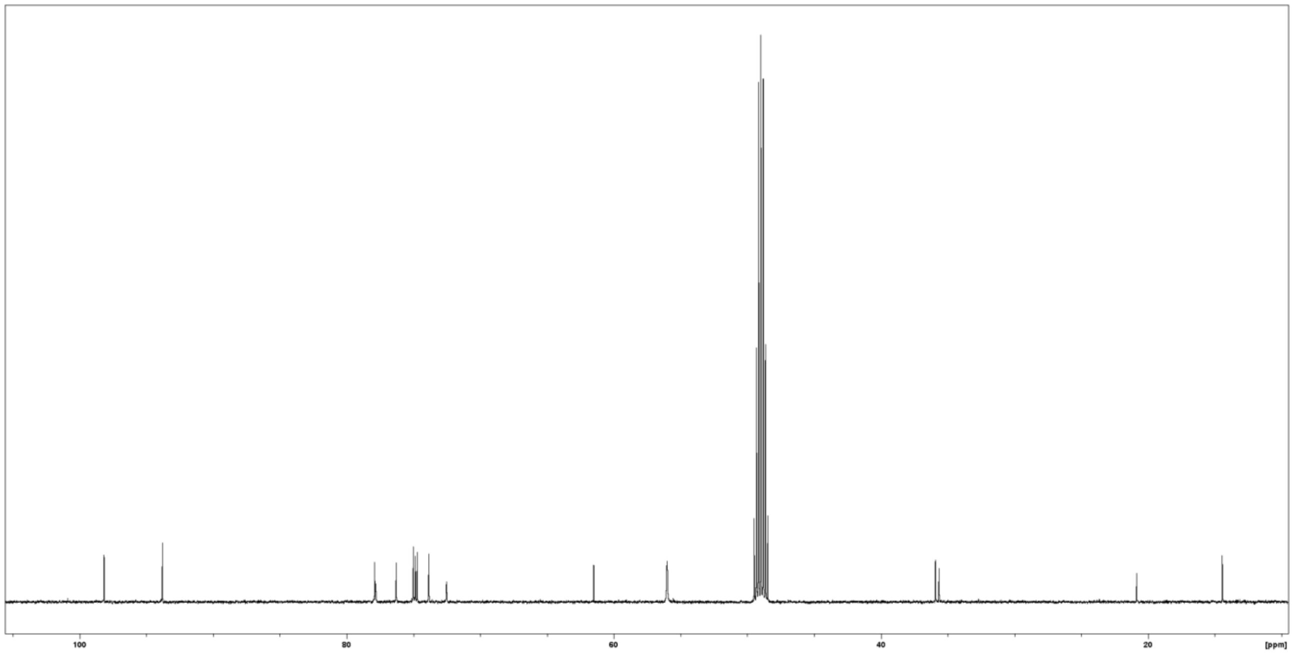

Figure S11. ^{13}C NMR spectrum of **4** (125.76 MHz, 25 °C, CD_3OD).

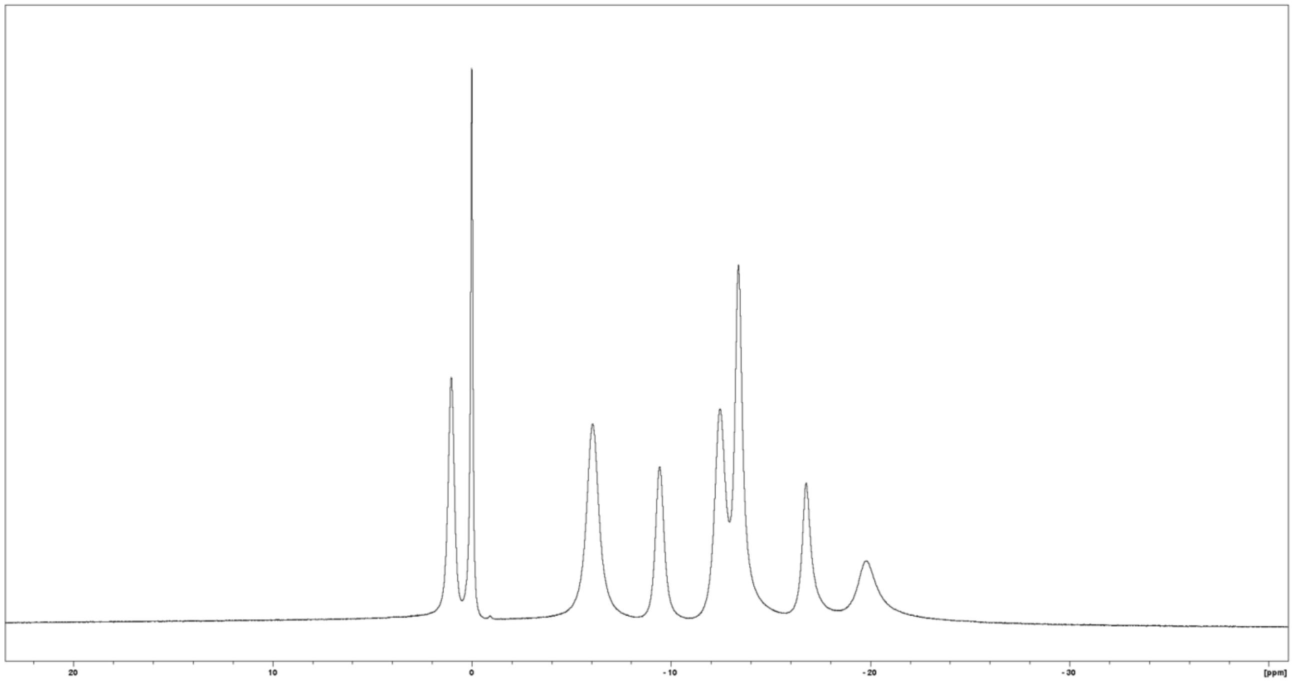

Figure S12. ^{11}B NMR spectrum of **4** (160.46 MHz, 25 °C, CD_3OD).

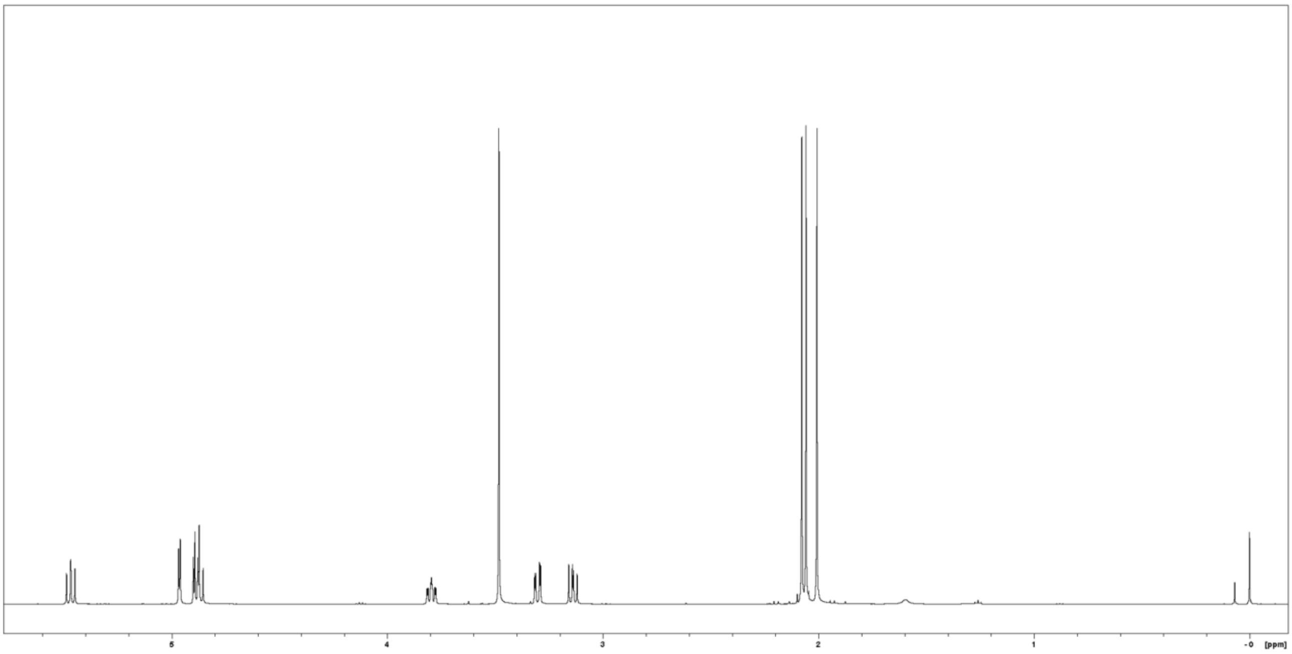

Figure S13. ^{1}H NMR spectrum of **5** (500.13 MHz, 25 °C, $CDCl_3$).

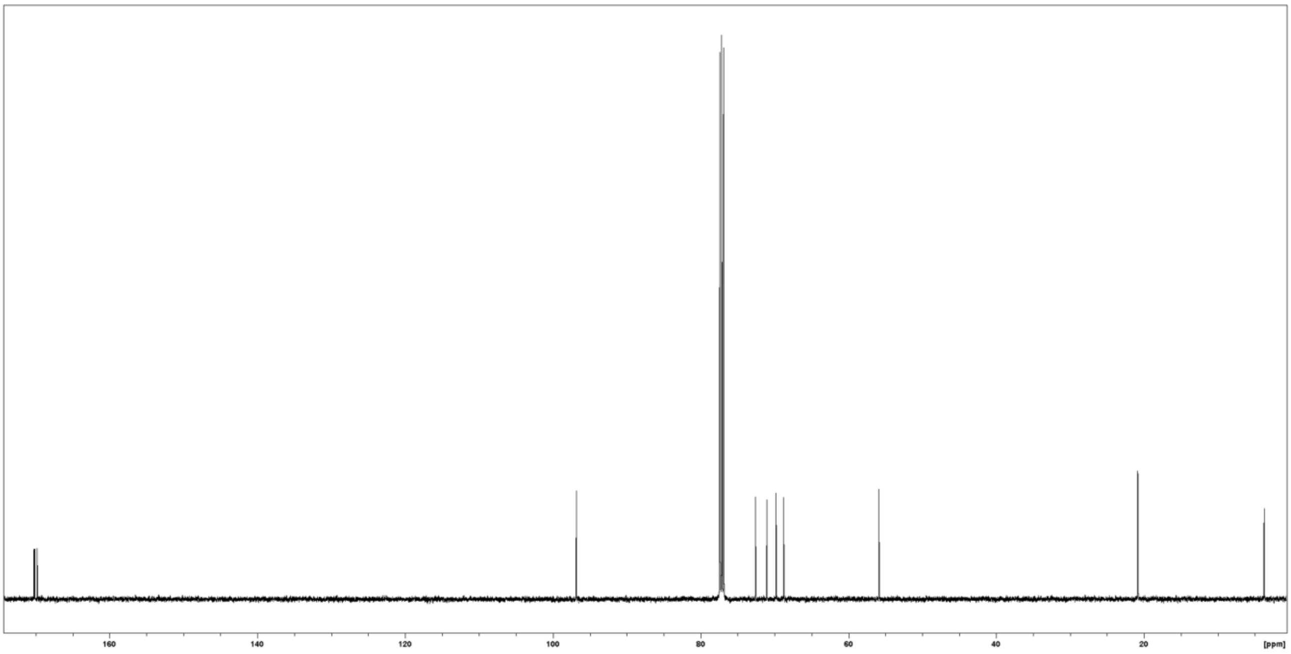

Figure S14. ^{13}C NMR spectrum of **5** (125.76 MHz, 25 °C, $CDCl_3$).

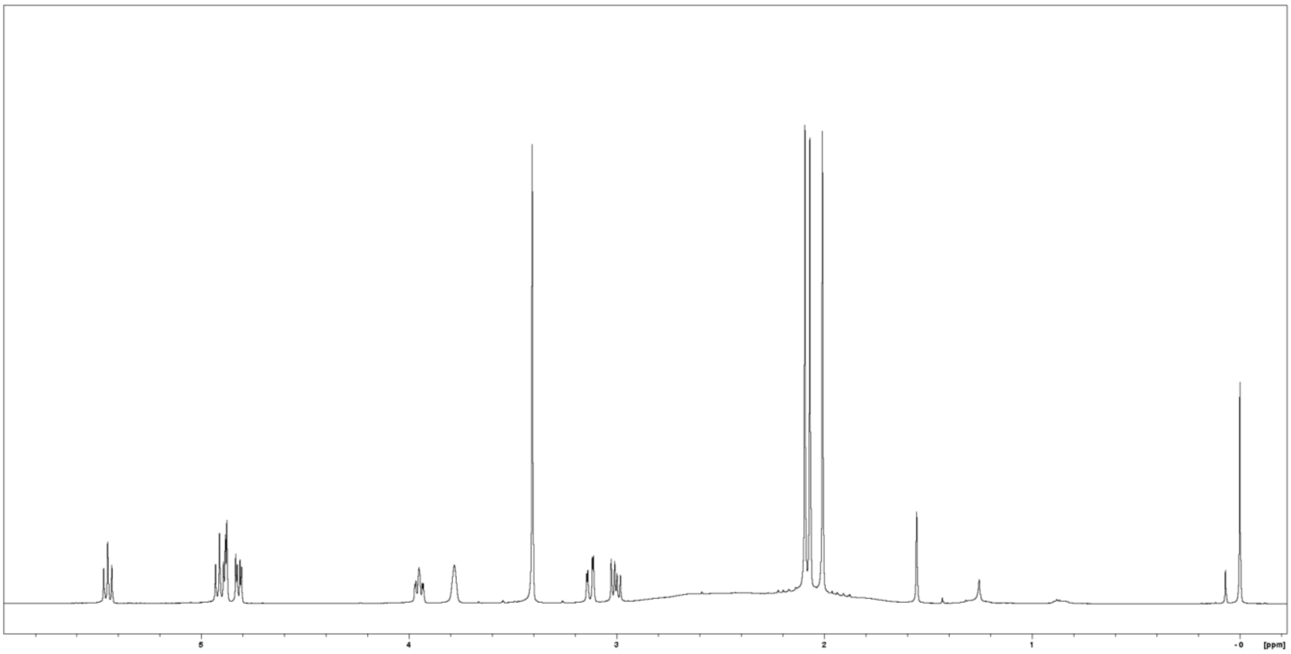

Figure S15. ^{1}H NMR spectrum of **6** (500.13 MHz, 25 °C, $CDCl_3$).

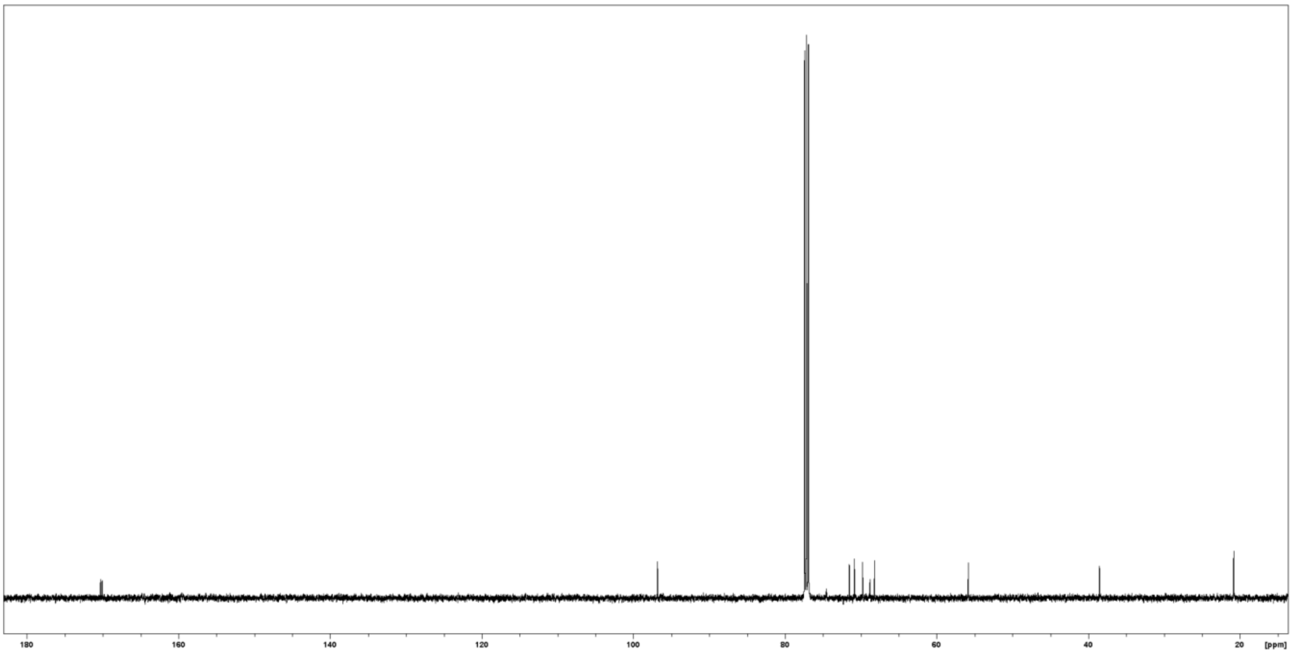

Figure S16. ^{13}C NMR spectrum of **6** (125.76 MHz, 25 °C, $CDCl_3$).

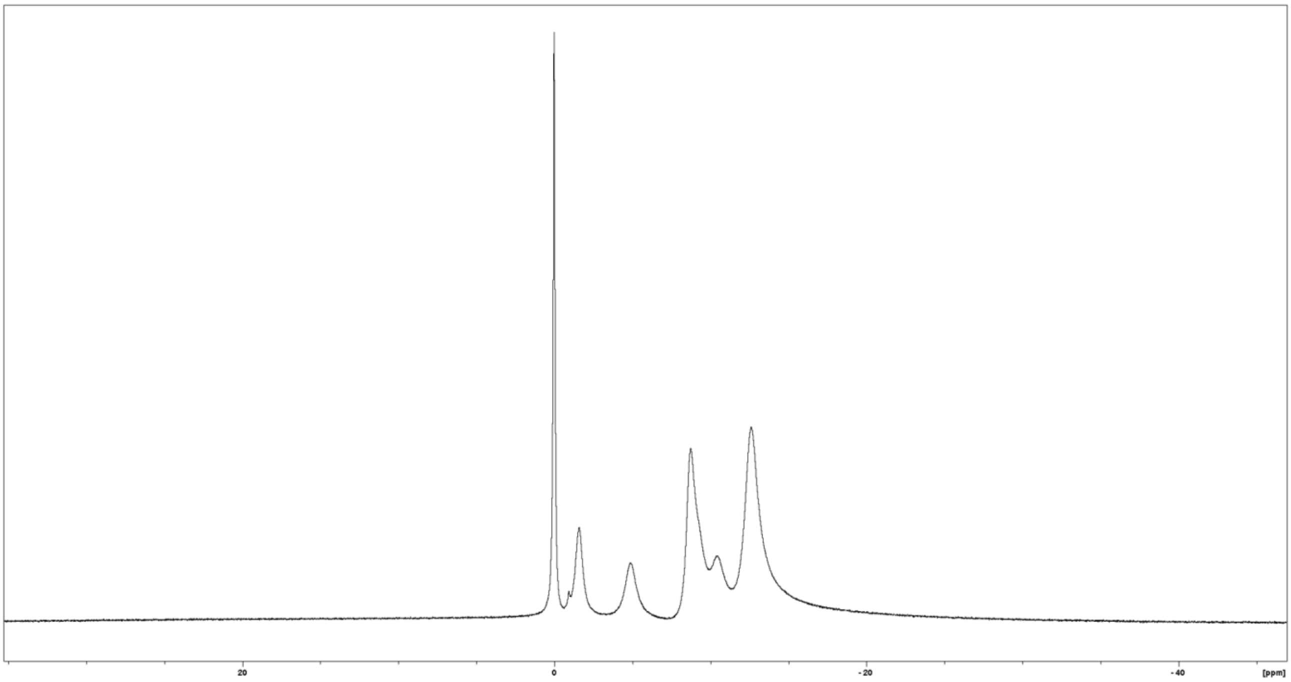

Figure S17. ^{11}B NMR spectrum of **6** (160.46 MHz, 25 °C, $CDCl_3$).

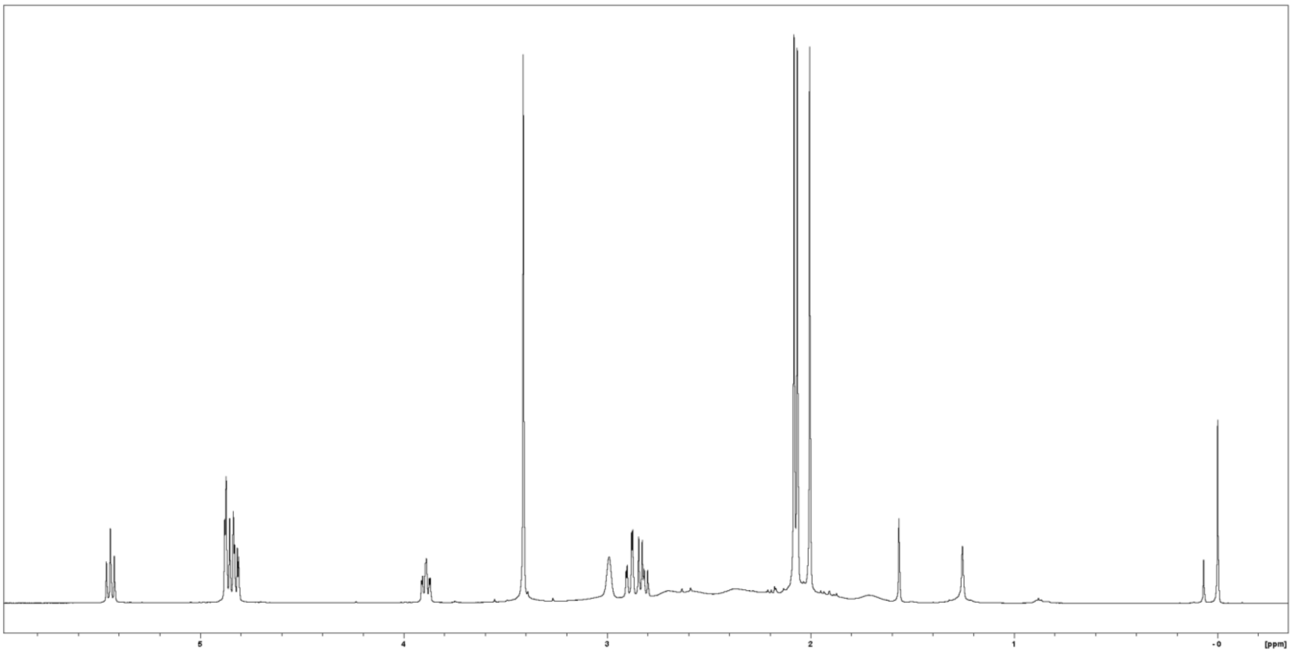

Figure S18. ^{1}H NMR spectrum of **7** (500.13 MHz, 25 °C, $CDCl_3$).

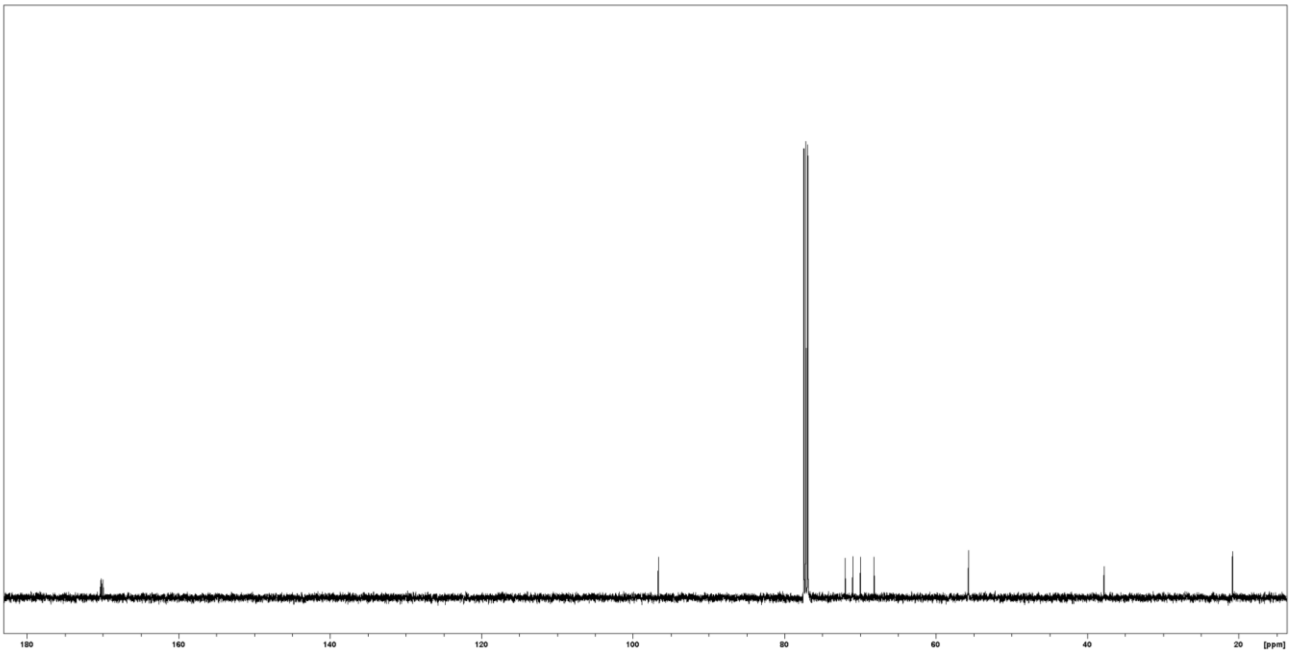

Figure S19. ^{13}C NMR spectrum of **7** (125.76 MHz, 25 °C, $CDCl_3$).

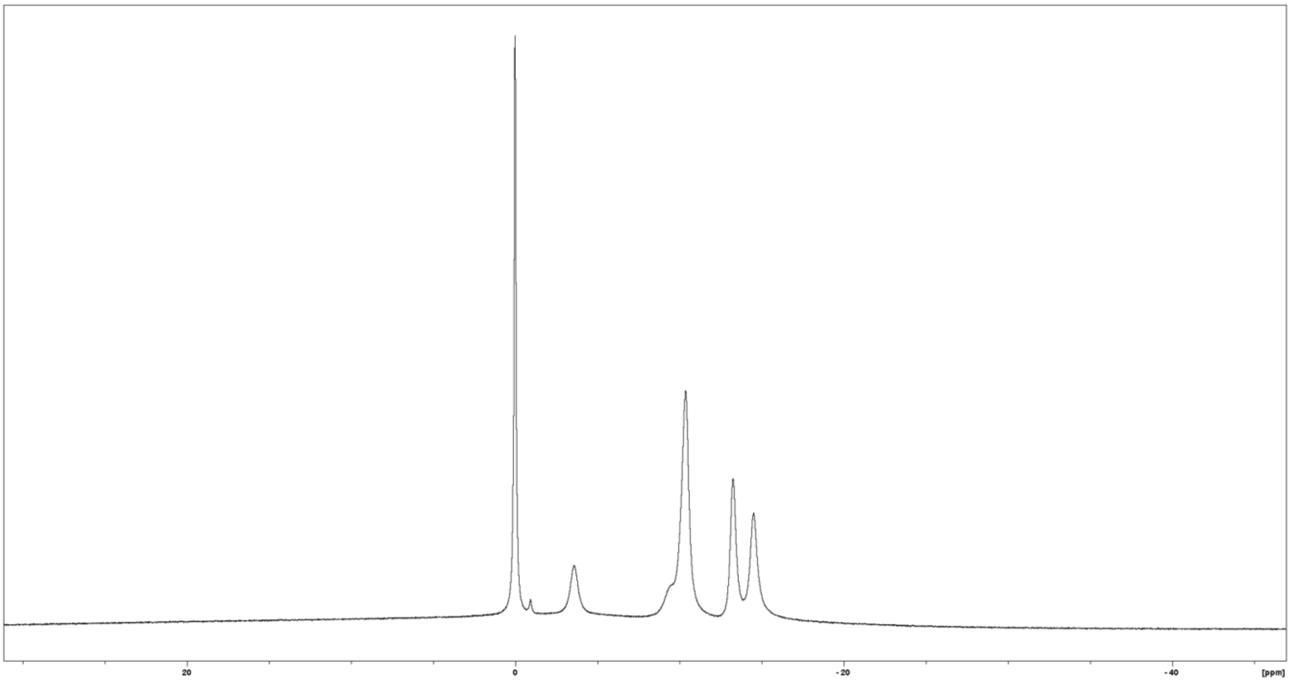

Figure S20. ^{11}B NMR spectrum of **7** (160.46 MHz, 25 °C, $CDCl_3$).

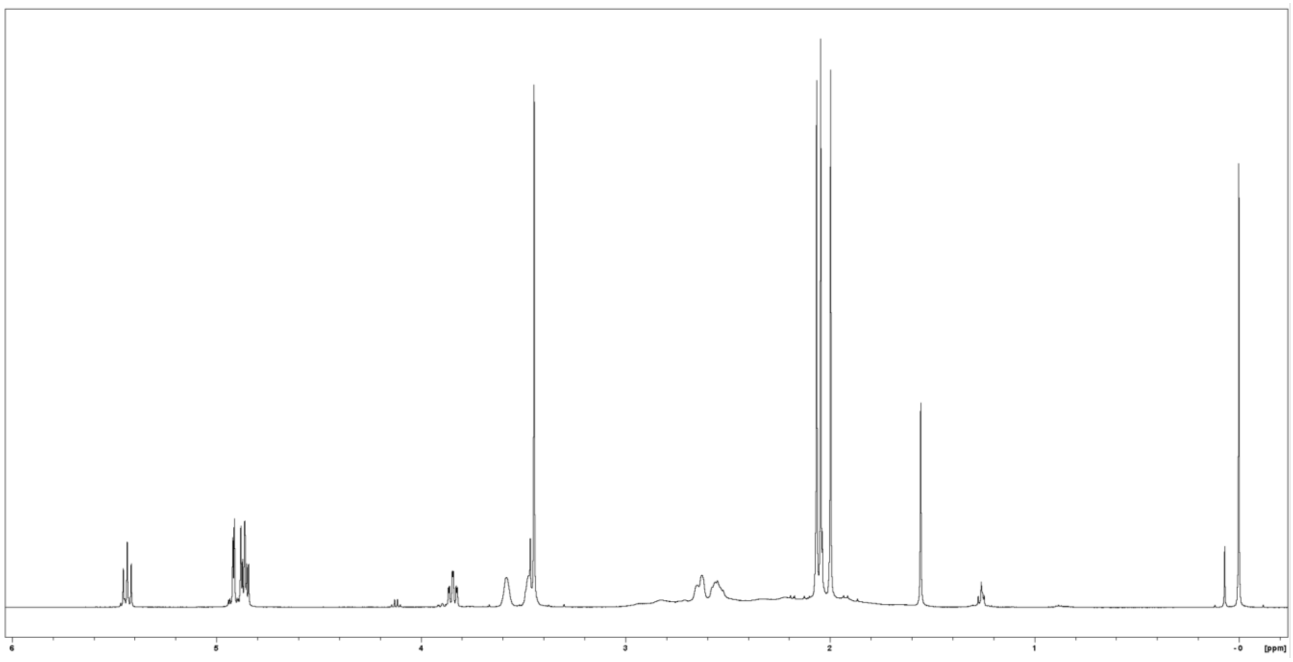

Figure S21. ^{1}H NMR spectrum of **8** (500.13 MHz, 25 °C, $CDCl_3$).

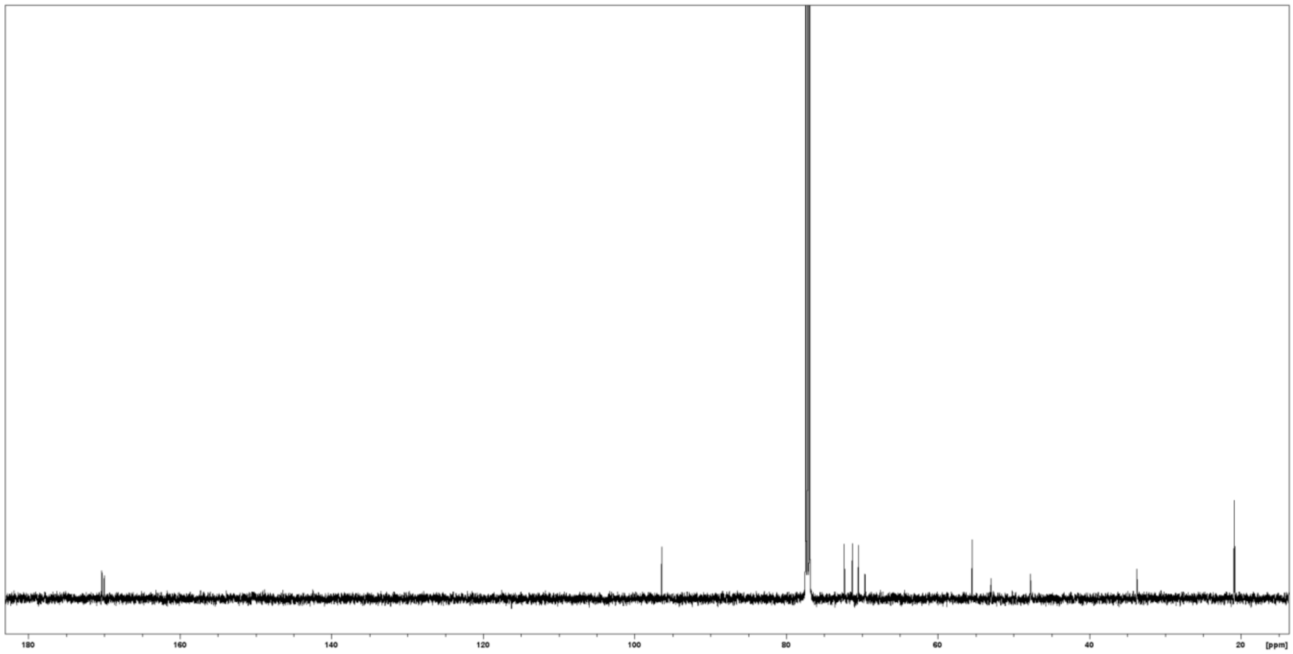

Figure S22. ^{13}C NMR spectrum of **8** (125.76 MHz, 25 °C, $CDCl_3$).

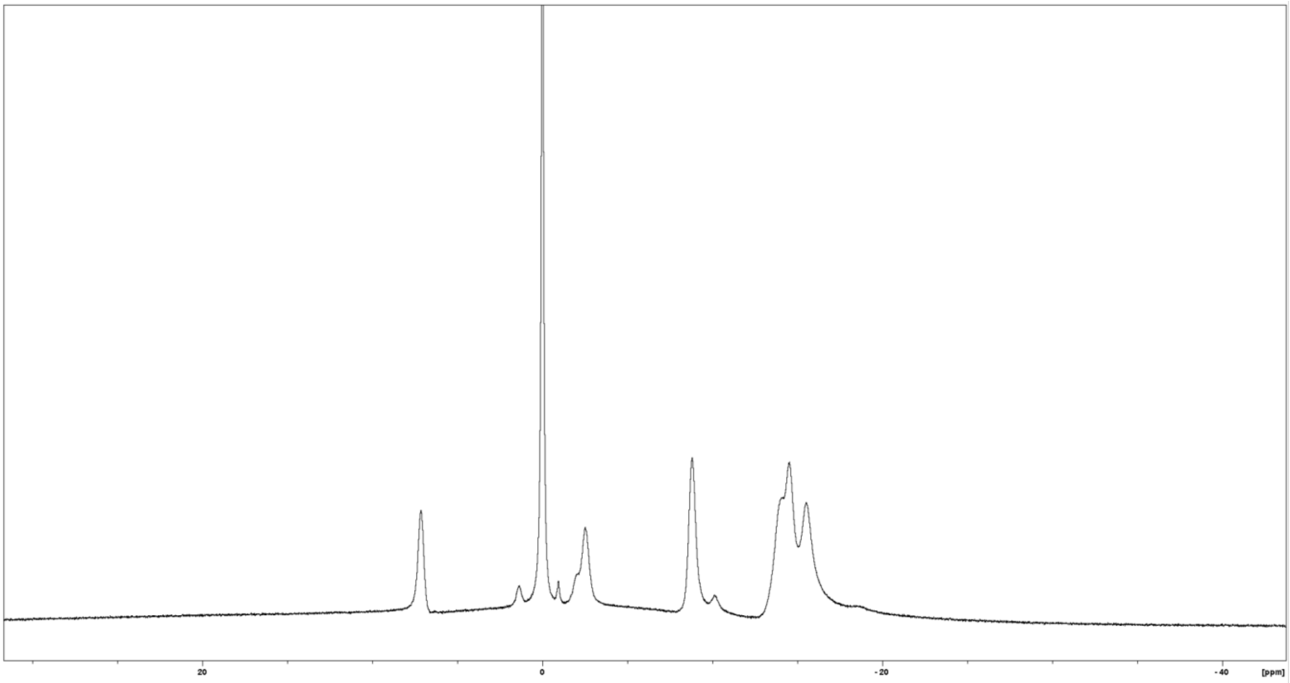

Figure S23. ^{11}B NMR spectrum of **8** (160.46 MHz, 25 °C, $CDCl_3$).

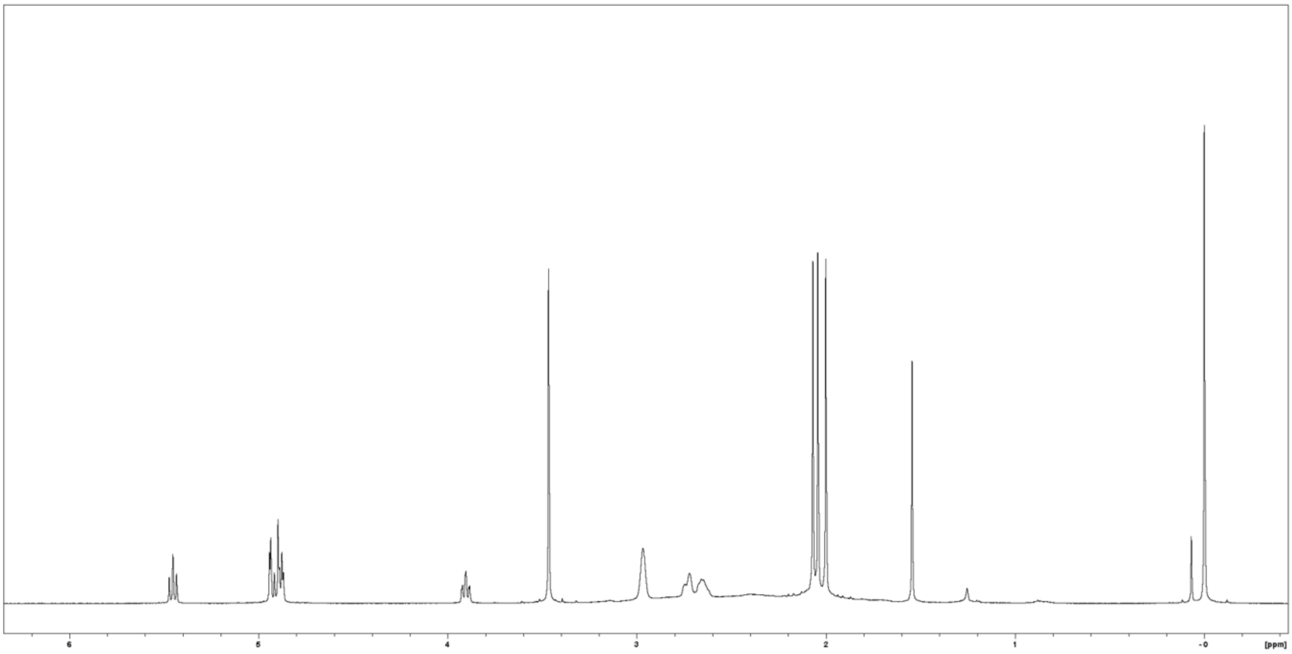

Figure S24. ^{1}H NMR spectrum of **9** (500.13 MHz, 25 °C, $CDCl_3$).

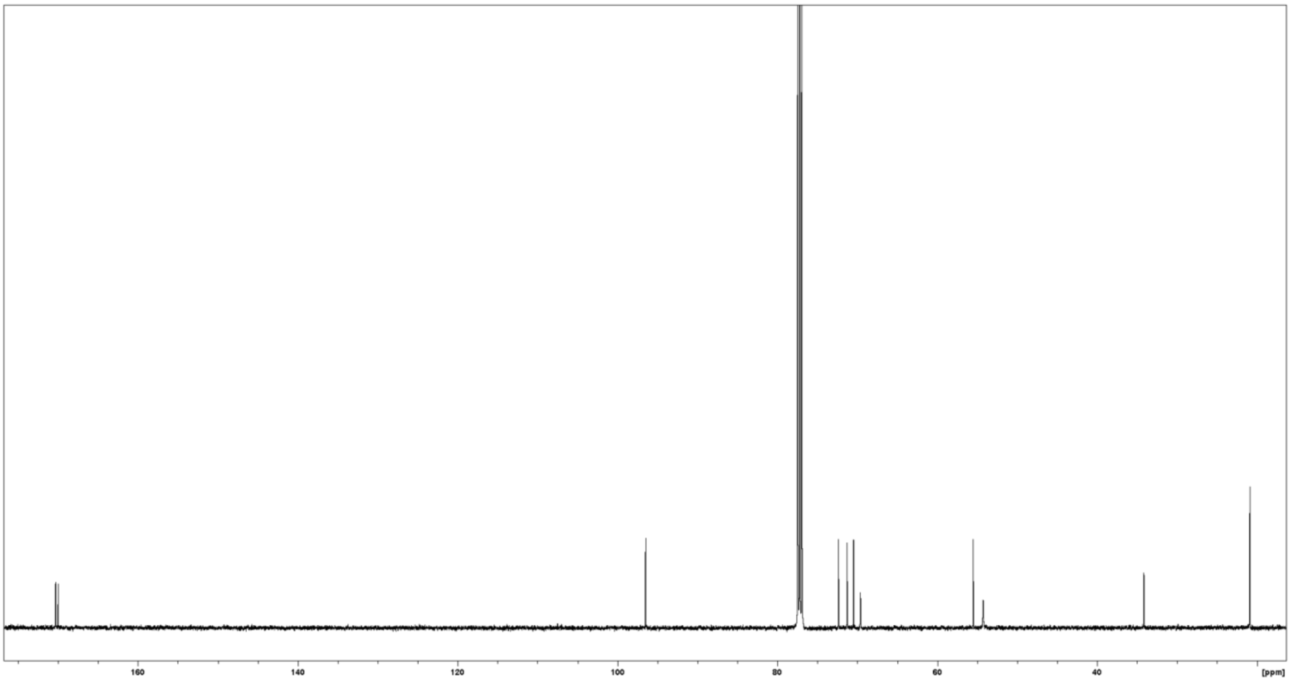

Figure S25. ^{13}C NMR spectrum of **9** (125.76 MHz, 25 °C, $CDCl_3$).

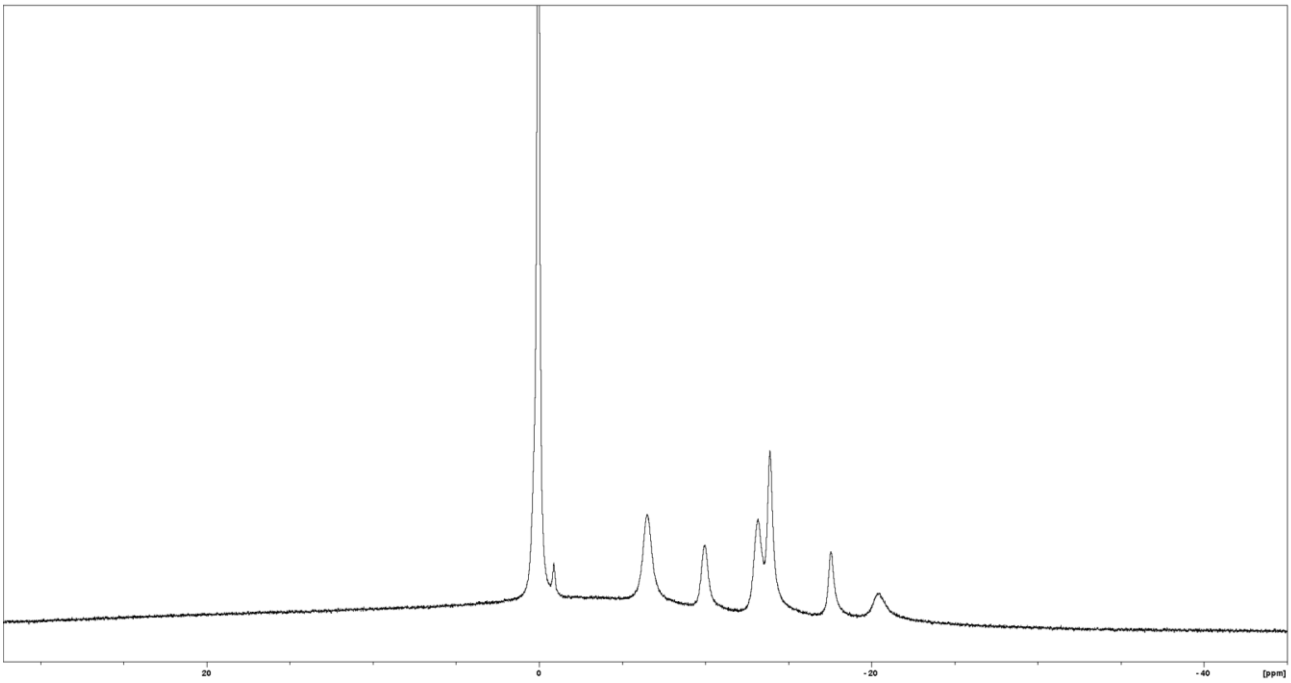

Figure S26. ^{11}B NMR spectrum of **9** (160.46 MHz, 25 °C, $CDCl_3$).

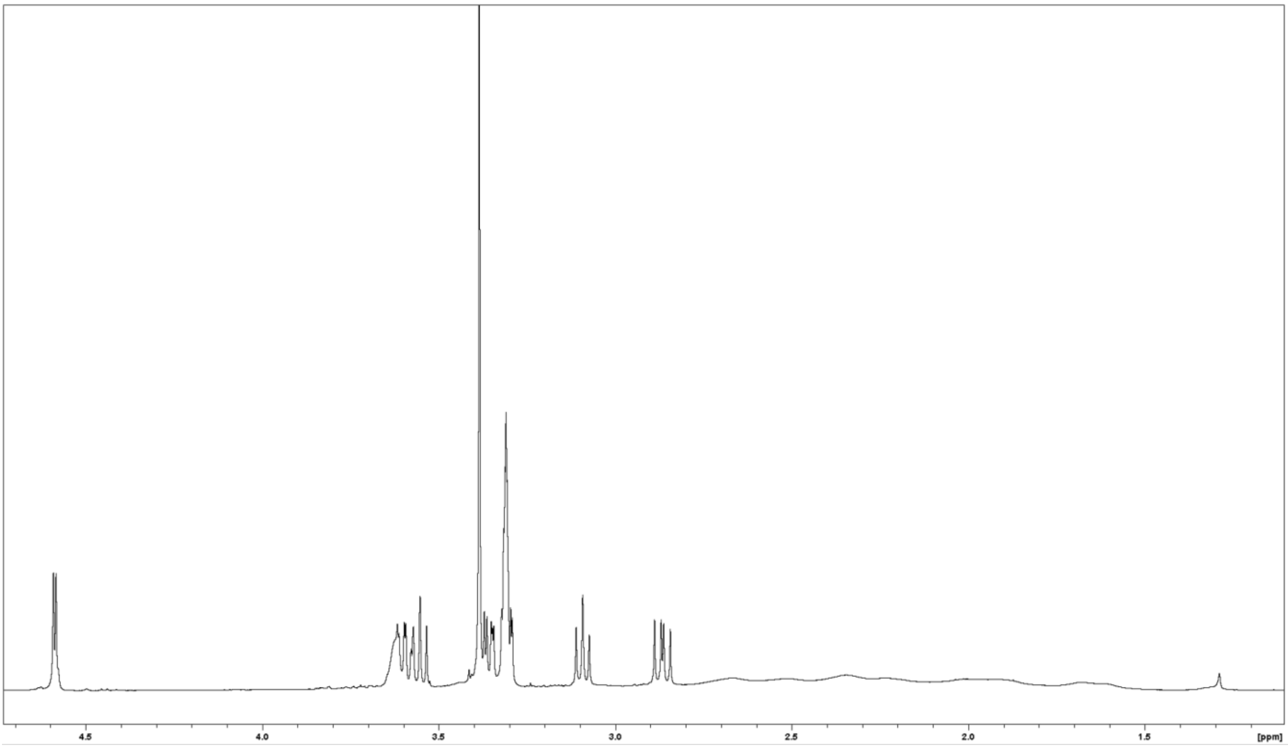

Figure S27. ^{1}H NMR spectrum of **10** (500.13 MHz, 25 °C, CD_3OD).

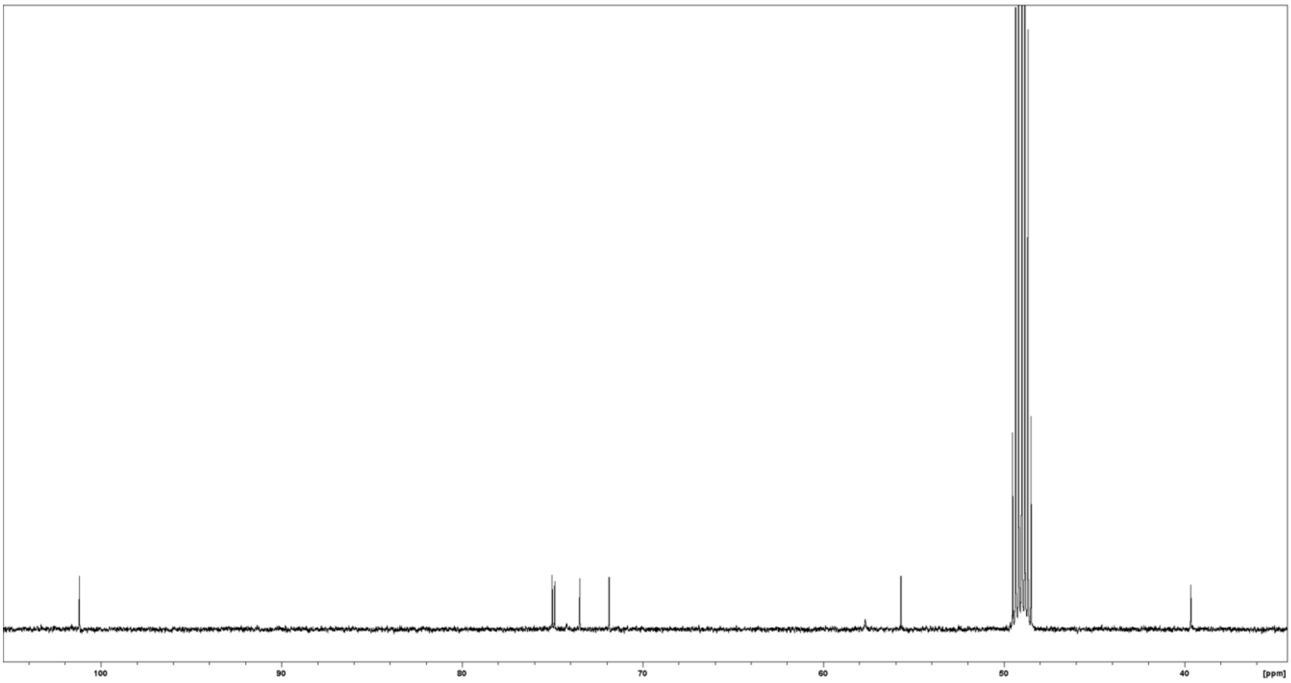

Figure S28. ^{13}C NMR spectrum of **10** (125.76 MHz, 25 °C, CD_3OD).

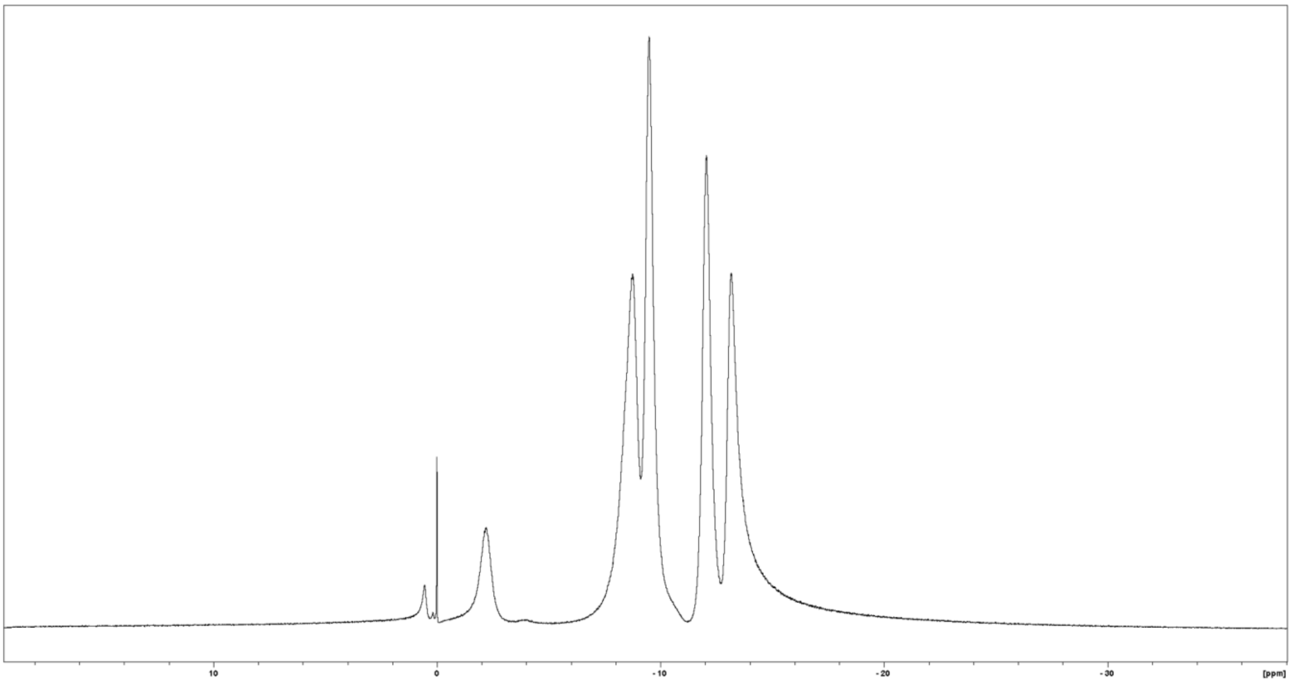

Figure S29. ^{11}B NMR spectrum of **10** (160.46 MHz, 25 °C, CD_3OD).

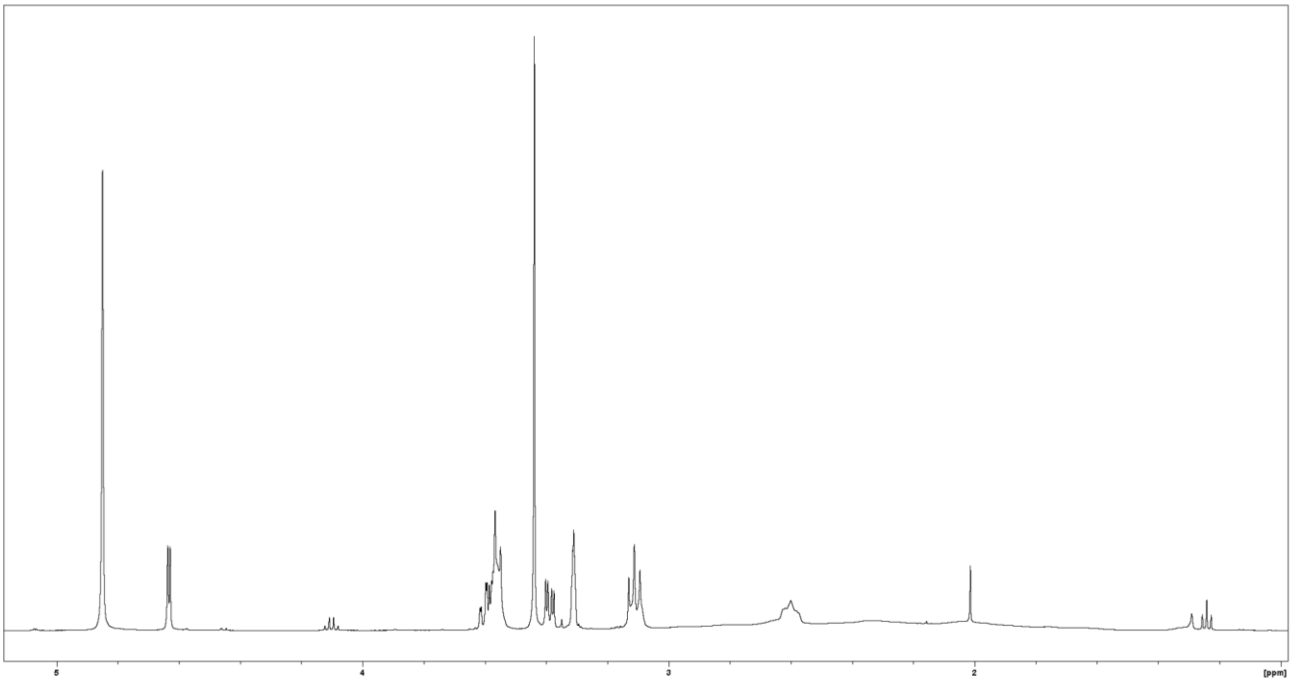

Figure S30. ^{1}H NMR spectrum of **11** (500.13 MHz, 25 °C, CD_3OD).

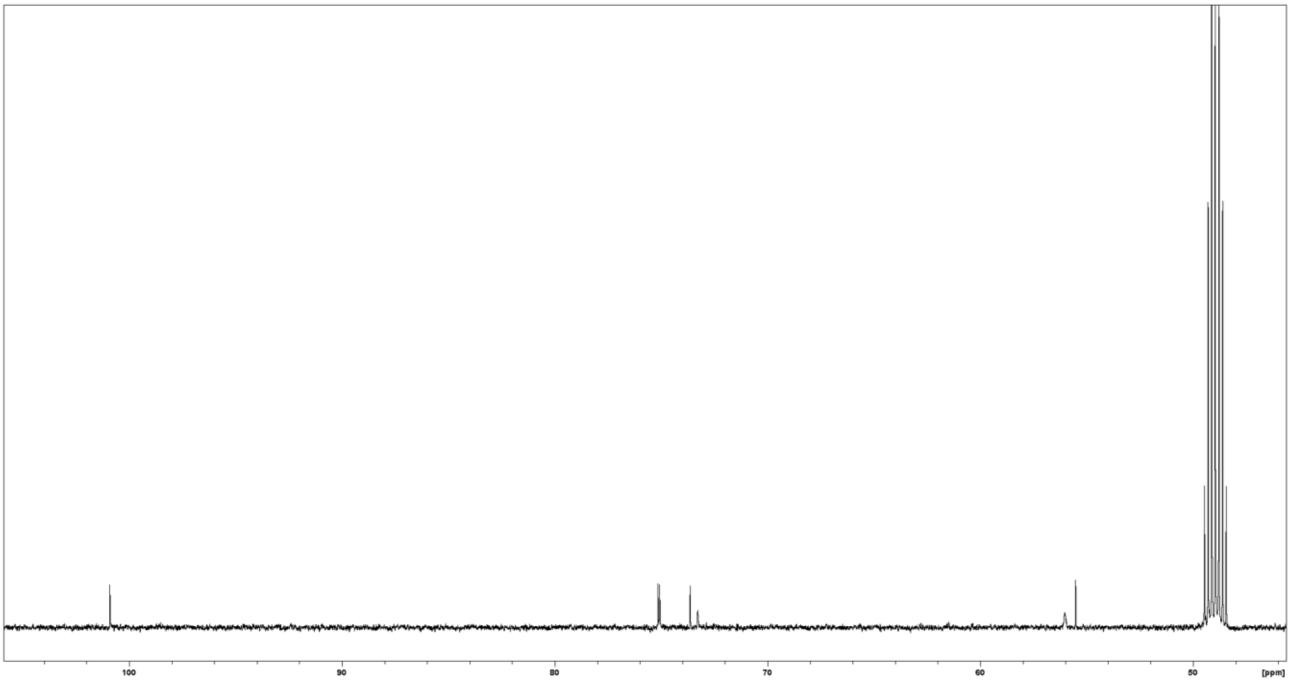

Figure S31. ^{13}C NMR spectrum of **11** (125.76 MHz, 25 °C, CD_3OD).

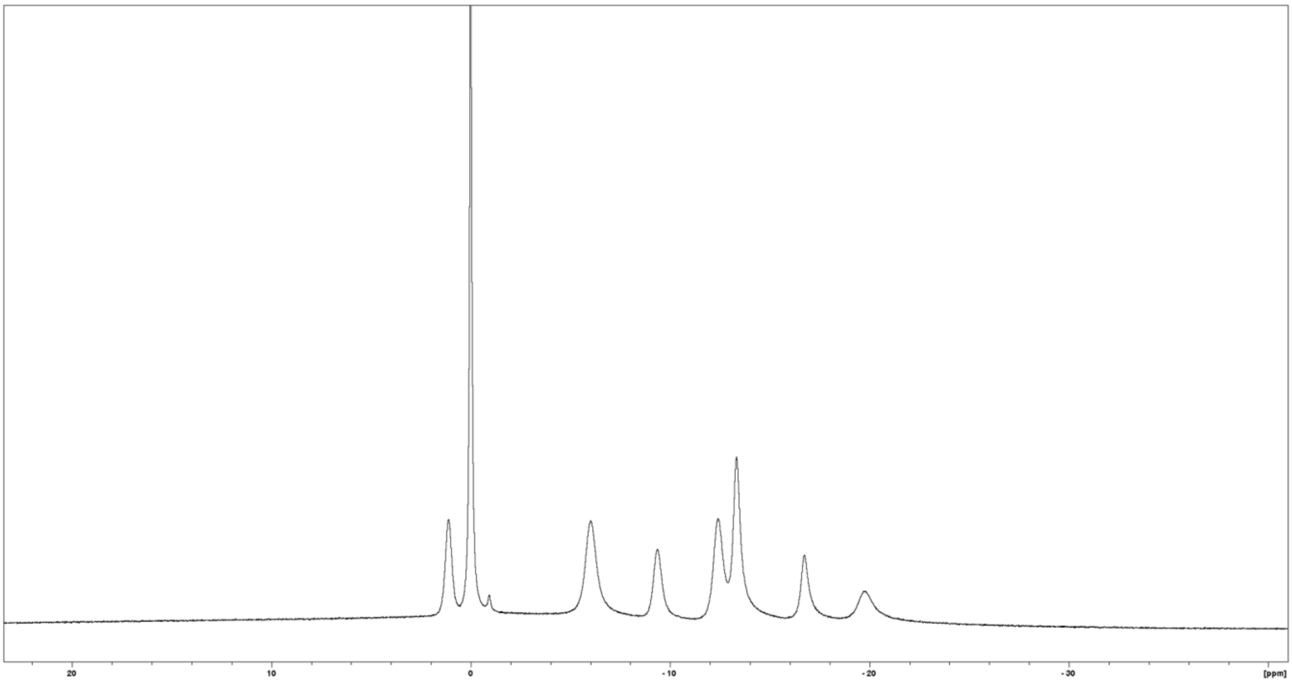

Figure S32. ^{11}B NMR spectrum of **11** (160.46 MHz, 25 °C, CD_3OD).

2. Molecular modeling

α and β anomers are indistinguishable in experimental studies, but computationally they can and were studied separately in the modeling assay. In order to compare the computational and experimental results, the overall mean binding energy for each α and β anomer was calculated and these were used to calculate the final mean binding energy for each glucoconjugate as a mixture of α:β (56:44 for **1**, 49:51 for **2**, 48:52 for **3**, and 55:45 for **4**). Thus the computational results can be directly compared to the computational results for D-glucose (α:β 36:64).

Table S1. Overall mean binding energy (MBE) of each glucoconjugate calculated as cluster weighted MBE where the α and β conformations of each ligand 1–4 has been calculated together. The ligands are in order from lowest binding energy to highest.

outward-open structure	mean binding energy (kcal/mol)	inward-open structure	mean binding energy (kcal/mol)
**1**	-5.92	**1**	-4.33
**2**	-5.80	**2**	-4.28
**4**	-5.17	**4**	-3.55
**3**	-4.93	**3**	-3.37
D-glucose	-0.61	D-glucose	-0.59
